# Sensor-Driven Surrogate Modeling and Control of Nonlinear Dynamical Systems Using FAE-CAE-LSTM and Deep Reinforcement Learning

**DOI:** 10.3390/s25165149

**Published:** 2025-08-19

**Authors:** Mahdi Kherad, Mohammad Kazem Moayyedi, Faranak Fotouhi-Ghazvini, Maryam Vahabi, Hossein Fotouhi

**Affiliations:** 1 Department of Computer Engineering and IT, University of Qom, Qom 46611, Iran; m.kherad@stu.qom.ac.ir; 2 CFD and Turbulence Research Lab., Department of Mechanical Engineering, University of Qom, Qom 46611, Iran; moayyedi@qom.ac.ir; 3 School of Innovation, Design and Engineering, Mälardalen University, 722 20 Västerås, Sweden; maryam.vahabi@mdu.se

**Keywords:** deep reinforcement learning, cyber-physical systems, sensor-driven modeling, control of dynamical systems, autoencoders, long short-term memory

## Abstract

**Highlights:**

**What are the main findings?**

- A surrogate-based deep reinforcement learning (DRL) framework (FAE–CAE–LSTM) is proposed.
- Achieves higher accuracy compared to DMD, POD, and conventional autoencoders.
- Provides 5–10× computational speedup, enabling real-time flow control.
- Incorporates a CNN–MLP reward estimator that enhances policy performance.

**What is the implication of the main findings?**

- Enables robust modeling of high-dimensional nonlinear flow dynamics.
- Reduces dependence on full-order PDE solvers, minimizing control delays.
- Scalable to turbulent and three-dimensional flow control problems.
- Offers broad applicability in fluid dynamics, energy systems, and engineering control.

**Abstract:**

In cyber-physical systems governed by nonlinear partial differential equations (PDEs), real-time control is often limited by sparse sensor data and high-dimensional system dynamics. Deep reinforcement learning (DRL) has shown promise for controlling such systems, but training DRL agents directly on full-order simulations is computationally intensive. This paper presents a sensor-driven, non-intrusive reduced-order modeling (NIROM) framework called FAE-CAE-LSTM, which combines convolutional and fully connected autoencoders with a long short-term memory (LSTM) network. The model compresses high-dimensional states into a latent space and captures their temporal evolution. A DRL agent is trained entirely in this reduced space, interacting with the surrogate built from sensor-like spatiotemporal measurements, such as pressure and velocity fields. A CNN-MLP reward estimator provides data-driven feedback without requiring access to governing equations. The method is tested on benchmark systems including Burgers’ equation, the Kuramoto–Sivashinsky equation, and flow past a circular cylinder; accuracy is further validated on flow past a square cylinder. Experimental results show that the proposed approach achieves accurate reconstruction, robust control, and significant computational speedup over traditional simulation-based training. These findings confirm the effectiveness of the FAE-CAE-LSTM surrogate in enabling real-time, sensor-informed, scalable DRL-based control of nonlinear dynamical systems.

## 1. Introduction

The control of nonlinear dynamical systems governed by partial differential equations (PDEs), such as those described by the Navier–Stokes equations, is critical for applications in energy, transportation, and healthcare. However, the complex, high-dimensional, and often chaotic dynamics of these systems make real-time feedback control computationally challenging. Reduced-order modeling (ROM) addresses this by capturing low-dimensional patterns from sparse sensor data (e.g., pressure or velocity measurements), enabling efficient control strategies.

ROM has emerged as a prominent area of research, driven by the goal of inferring efficient dynamical models from data. This domain has witnessed the development of a diverse repertoire of techniques for data-driven dynamical model learning, encompassing established methods such as balanced truncation and proper orthogonal decomposition (POD) alongside more recent advancements like dynamic mode decomposition (DMD) [1]. There are two primary classifications of ROM methods, distinguished by their dependence on governing equations: intrusive ROM models (IROMs) and non-intrusive ROM models (NIROMs) [2]. IROMs are dependent on the governing equations and source code. Due to their intrusiveness, they preserve many of the physical characteristics from the original system [3]. Two fundamental challenges in IROMs are stability [4] and nonlinear efficiency [5]. Additionally, changing the source code of IROMs is difficult [6]. NIROMs do not require any knowledge of the physical systems. One of the reasons for the development of NIROMs is that in most cases, the source code describing the physical model must be modified to produce the reduced-order model, and these changes may be complex, especially in old codes, or may not be possible if the source code is not available [3].

A recent trend in control theory involves the integration of ROM techniques with optimal and stable control strategies to develop controllers for nonlinear dynamical systems [7,8,9]. As a primary ROM method, POD is widely used in control. DMD has emerged as another frequently employed technique within the realm of ROM for flow field analysis and control applications in recent years.

On the other hand, within the domain of machine learning control for complex systems, a significant focus has been placed on learning-based approaches. Model-free control strategies, which aim to directly learn control policies without explicit environment modeling, have achieved notable success across various applications. However, these methods often necessitate extensive interaction with the environment to acquire effective policies, rendering them potentially impractical for scenarios where simulations are computationally expensive. Conversely, model-based control approaches offer the potential to learn efficient controllers with significantly less data through the initial establishment of an environment model. While successful in certain domains, model-based methods can be computationally demanding themselves [10,11], but such success requires building accurate dynamic models.

Deep learning, a recent innovation in artificial neural networks, empowers the extraction of increasingly intricate patterns from data. This technology excels at processing raw, unprocessed data and leverages a multi-layered architecture to train a nonlinear system. This capability allows deep learning models to not only represent complex relationships within data but also make predictions based on these learned patterns [12]. Deep learning includes many neural networks such as convolutional neural networks (CNNs), recurrent neural networks (RNNs), and autoencoders [13]. The core architecture of a CNN comprises three fundamental layer types: convolutional, pooling, and fully-connected layers [14]. Autoencoders [15] are neural networks that compress high-dimensional data into a low-dimensional latent space (encoding) and reconstruct it (decoding). Convolutional autoencoders (CAEs) use convolutional layers to capture spatial patterns, while fully connected autoencoders (FAEs) handle nonlinear feature extraction. LSTMs are a type of RNN designed to model sequential data, using gating mechanisms to capture long-term temporal dependencies in dynamic systems [16]. Today, deep learning is also used as one of the important solutions in the field of ROM and control.

For system control, reinforcement learning (RL) is an advanced method. RL is self-organized learning, where learning is achieved through experience and the outcome of the experience [17]. That is, RL collects the data itself and learns from it. In summary, for RL, the agent provides the actuators to the environment in order to maximize the yield or cumulative reward, based on the system’s state. In RL, improving the actuator action creates a mathematical problem for an agent (controller), where the relationship between the reward in terms of forces caused by the fluid and the action is not specified. Therefore, to approximate the relationship, the agent is replaced with a deep learning network, hence it is called deep reinforcement learning (DRL) [18]. DRL presents itself as a promising avenue for tackling control problems in fluid dynamics. However, a significant challenge persists: constructing accurate models using computational fluid dynamics (CFD) simulations often necessitates substantial computational resources and extended execution times.

This paper addresses the following core research question: Can we achieve real-time control of nonlinear dynamical systems using only sparse sensor data, without access to full-order models or governing equations, by leveraging deep learning-based surrogate modeling and reinforcement learning?

To this end, the proposed method for controlling a dynamic system is a combination of DRL and data-driven ROM, which is used to learn control policies from a limited dataset and does not require the governing equations and control expressions to be known. Many high-dimensional systems actually exhibit low-dimensional dynamics, and the goal of the proposed method is to extract and model these dynamics from data using a combination of autoencoders and LSTM. Then, control policies are extracted from the proposed data-driven ROM using DRL. It is important to note that the proposed method does not require RL to directly interact with the target system, and it also does not require significant changes to standard RL algorithms. In flow control, distributed pressure sensors and velocity probes yield sparse, high-dimensional data streams. Processing this data in real-time using full-order simulations is infeasible. Our method enables control policy learning from such sensor-acquired states. In this research study, a data-driven NIROM, called FAE-CAE-LSTM, is proposed. The core contributions of this work are summarized as follows:

- A novel sensor-driven surrogate modeling approach is introduced by combining FAE, CAE, and LSTM networks to capture both spatial and temporal dynamics of nonlinear dynamical systems from sparse measurements.
- An integrated model-based DRL control framework is proposed, which allows the policy to be trained entirely in the latent space of the reduced-order model, significantly reducing computational cost and training time.
- A data-driven reward estimator based on CNN–MLP architecture is developed to approximate the reward function from sensor-like inputs, eliminating the need for access to full-order simulation or physical models during training.
- The proposed approach is general, non-intrusive, and scalable, making it suitable for a wide range of physical systems governed by PDEs, without requiring explicit knowledge of the governing equations.
- Extensive validation is conducted on multiple benchmark PDE systems (e.g., Burgers’, Kuramoto–Sivashinsky, and flow past cylinder), demonstrating superior performance compared to existing DRL-based control methods.

In the following section, we review the most recent applications of DRL in the control of dynamic systems. Then, in the third section, the proposed method for controlling a dynamic system is given. The obtained results are presented in the fourth section and finally, the conclusion is presented in the fifth section.

## 2. Related Works

In recent years, several machine learning-based frameworks have been proposed for the ROM of nonlinear dynamical systems using autoencoders and recurrent architectures. For example, Simpson et al. [19] introduced a hybrid architecture combining autoencoders and LSTM networks for model order reduction, highlighting their ability to capture nonlinear temporal dynamics. Similarly, Drakoulas et al. [20] presented FastSVD-ML-ROM, which integrates machine learning and singular value decomposition for real-time surrogate modeling. Maulik et al. [21,22] explored both autoregressive and non-autoregressive schemes using CAE and RNNs to model advection-dominated systems, emphasizing stability and generalization. Mücke et al. [23] proposed a memory-aware deep learning framework for time-dependent PDEs, showing improved prediction quality in parameterized settings.

Several studies have examined the use of DRL for the control of complex, nonlinear, and even chaotic systems. Vignon et al. [24] provide a comprehensive review of recent DRL techniques in flow control, outlining their advantages and limitations. Bucci et al. [25] demonstrated the ability of DRL to stabilize chaotic dynamics, showing promising results on benchmark systems. Zolman et al. [26] introduced the SINDy-RL framework, which combines interpretable model-based learning with DRL for more efficient and explainable policy generation.

Recent advances have demonstrated the effectiveness of DRL in controlling complex dynamical systems, particularly in fluid mechanics. DRL has been successfully applied to tasks such as vortex shedding suppression, flow stabilization, and thermal control. Its ability to discover high-level control strategies aligns well with the closed-loop nature of feedback control. Applications extend across various domains including UAV control [27], glider flight path optimization [28], and shape optimization [29,30], where flow features like pressure or velocity fields define the system state and control actions aim to minimize drag or mitigate instabilities.

Rabault et al. [31] demonstrated the use of DRL for flow control around a cylinder using air jets and pressure sensors, achieving 8% drag reduction with the proximal policy optimization (PPO) algorithm. They also introduced the parallelization of CFD simulations to significantly accelerate training. Similarly, Tokarev et al. [32] employed PPO for flow control using cylinder rotation, exploring different reward functions and reporting up to 16% drag reduction with stable behavior.

Paris et al. [33] applied a modified PPO algorithm (S-PPO-CMA) for flow control using suction and blowing near a cylinder, achieving 4.18% drag reduction. Their study further examined the impact of sensor placement and quantity on DRL performance. An improved version, AS-PPO-CMA, was later proposed to reduce actuator usage with minimal performance loss [34]. In a related work, Tang et al. [35] employed PPO with multiple synthetic jets and demonstrated significant drag reduction across various Reynolds numbers, reaching up to 73.8%.

To overcome the limitations of conventional DMD in handling external control inputs, DMD with control (DMDc) was introduced [36]. Building on this, [37] integrated DMDc with DRL for flow control around a circular cylinder. By using DMDc to extract spatiotemporal features from flow snapshots, they designed a data-driven reward function based on mode amplitudes, leading to effective wake vortex stabilization and improved DRL performance.

Drag reduction is by far the most widely used application domain in DRL control of computational dynamical systems. Almost all studies in this area have focused on two-dimensional (2D) incompressible flows around cylinders (typically different sections of a cylinder) subjected to uniform or parabolic velocity profiles, and have used model-free DRL methods [31,32,33,35,37,38,39,40,41,42,43,44,45,46,47,48,49]. Most DRL algorithms used in this field are of the actor–critic (AC) type, due to the use of the pre-implemented PPO algorithm in packages such as Tensorforce [50], OpenAI baselines [51], or Stable Baselines [52]. In terms of CFD solvers, FeniCS has emerged as the most widely used tool, as the source code of the work by Rabault et al. [31] has been heavily reused in subsequent works. With regard to flow patterns, almost all works consider laminar (non-turbulent) conditions with Reynolds numbers in the range of hundreds to a few hundreds. Regarding the numerical reward function, most works in the field of drag reduction are based on the scheme proposed in [38]:

(1) $$r_{t}=-\langle C_{D}\rangle -\beta |\langle C_{L}\rangle |$$

where the <.> operator denotes the sliding average over a time period of T wake or a single action time step. *C_D_* and *C_L_* are, respectively, the drag and lift coefficients. The parameter β varies in the range of 0.2 to 1 in the papers and prevents the network from achieving drag reduction based on a large lift force. Recently, the use of DMD to design the reward function based on mode amplitudes, has led to efficient control strategies [37]. Finally, the architectures of the actor networks are also similar in different studies, with two fully connected layers in all cases. While a number of papers use very large layer sizes, smaller networks have been used successfully for problems of similar dimensions. In fact, no large-scale study has been conducted on the impact of network architecture on the performance of the final agent, and this choice often remains experimental.

Model-based DRL has so far been used primarily for relatively simple environments. Sun et al. [53] introduce a novel approach that combines Gaussian process regression (GPR) with PPO for a pendulum control task. This method achieves performance comparable to standard PPO while requiring fewer interactions with the environment. However, a key limitation of GPR lies in its computational inefficiency when dealing with high-dimensional data [54]. Consequently, its application to most fluid mechanics problems remains impractical due to the inherently high-dimensional and complex nature of fluid flow data, which would render GPR computationally intractable. In addition, Kaiser et al. [55] presented a model-based DRL approach for training an agent to play Atari games. This approach leverages video prediction using CNNs as the underlying model.

Chung et al. [56] present a novel multi-agent RL approach to efficiently sample solutions for a class of challenging multiscale inverse problems by formulating it within the RL framework. It demonstrates faster convergence compared to standard Monte Carlo Markov Chain (MCMC) methods.

Bieker et al. [57] developed a model predictive control (MPC) framework incorporating a deep RNN to learn dominant flow patterns from sensor data. Applied to two-dimensional flow around a cylinder, the RNN served as a surrogate model for real-time control, successfully predicting lift and drag forces under varying CFD conditions.

Recently, kurz et al. [58] formulated eddy viscosity modeling in large eddy simulations as an RL task, using a CNN-based policy to adapt viscosity based on local flow states. Their approach yielded stable and accurate long-term simulations. Extending this work, they introduced Relexi [59], a scalable RL-CFD framework designed for high-performance computing (HPC) environments, enabling real-time interaction between RL agents and CFD solvers for improved turbulence modeling.

Mao et al. [60] proposed DRLFluent, a distributed framework for active flow control (AFC) that connects DRL agents with CFD solvers across different nodes via a broker architecture. The framework achieved notable drag reduction and scalability, especially with over 20 parallel environments. However, its reliance on HPC infrastructure may limit accessibility for some users.

Guastoni et al. [61] introduced a DRL environment for designing and evaluating control strategies aimed at reducing drag force in confined turbulent fluid flow in a channel. The environment provides a framework for computationally efficient, parallelized, and high-quality fluid simulations, with the ability to interface with RL agent programming interfaces.

Xu and Zhang [62] evaluated a model-free DRL approach for controlling convective flows under random disturbances, starting with the Kuramoto–Sivashinsky equation and extending to two-dimensional boundary layer flows. They emphasized optimal sensor placement to enhance control efficiency.

Wang et al. [63] introduced a two-stage DRL method for drag reduction around a cylinder, combining CNN-based feature extraction from pressure signals with a hierarchical neural network (HNN) controller, demonstrating robust performance across varying flow conditions.

In recent years, several studies have addressed the integration of deep learning with sensor-based inputs for control and inference in complex environments. Marsh et al. [64] provide a comprehensive review of deep learning-based sensor fusion techniques, emphasizing the potential of end-to-end trainable models to enhance robustness and accuracy in multi-sensor environments.

Liu et al. [65] introduced a CNN-based transmitter localization approach using sparse wireless sensor networks. Their method demonstrated that deep learning can accurately infer system state from highly limited sensor inputs, a principle directly relevant to surrogate modeling in sparse sensor fields.

In the context of control and predictive maintenance, Skordilis et al. [66] proposed a DRL framework for sensor-driven decision-making in real time. By combining DRL with particle filtering, their approach achieved robust control in environments with uncertain and noisy sensor data.

Furthermore, Fukami et al. [67] explored global field reconstruction using Voronoi tessellation-assisted deep learning. This work highlights how surrogate models can be trained from sparse spatial sensors to accurately recover high-dimensional flow fields—an idea closely aligned with the objective of the FAE-CAE-LSTM model proposed in this paper.

These sensor-driven AI methods affirm the relevance of combining deep learning and sparse measurements to enable scalable, real-time control strategies for dynamical systems. Chen et al. [68] proposed a secure sensor fusion algorithm for nonlinear autonomous vehicle control under sensor attacks, highlighting the impact of data reliability on control performance, which is critical for sensor-driven approaches.

Rabet et al. [69] developed ACTOR, a DRL system for real-time transmission power control in wireless sensor networks, demonstrating DRL’s applicability to embedded control, similar to our proposed DRL framework for nonlinear dynamical systems.

Abbaspour Gildeh et al. [70] presented a comparative study of hybrid deep learning models for human activity recognition using wearable and ambient sensors. The study highlights how convolutional and RNN combinations can effectively capture spatial and temporal sensor data correlations, which parallels our use of CAE–FAE–LSTM for modeling spatiotemporal dynamics from sensor-like flow snapshots.

Physics-informed deep learning (PIDL) has emerged within scientific machine learning (SciML) as a powerful approach that incorporates physical laws into deep learning to improve generalization and data efficiency. Building on this idea, Liu and Wang [71] proposed physics-informed model-based reinforcement learning (PiMBRL), which integrates physics-based constraints into the RL training process. Their framework uses an autoencoder-based recurrent architecture and off-policy actor–critic algorithms to reduce model bias and reliance on real-world data, thereby enhancing learning efficiency in complex dynamical systems.

Sun and Thiagarajan [72] introduced a multi-fidelity RL framework that combines a coarse physics-based model with sparse high-fidelity corrections. This approach enabled reliable DRL policy learning for chaotic physical systems at a fraction of the computational cost of high-resolution simulations.

Compared to prior DRL-based control approaches, our method introduces several distinctive advantages:

- Surrogate modeling: While many studies use POD, DMD, or CNN-only models, we employ a hybrid FAE–CAE–LSTM structure that jointly captures spatial and temporal features from sparse sensor data.
- Latent-space control: Unlike methods requiring interaction with full-order CFD simulators during training, our DRL agent is trained entirely in the reduced latent space, significantly reducing computational cost.
- No access to physical models: Prior works often assume known governing equations or ground-truth reward functions. In contrast, we use a CNN–MLP-based reward estimator that approximates reward signals directly from sensor-like input, making our framework more adaptable and non-intrusive.
- Generalizability: Our framework is validated across various PDE systems, unlike many existing methods focused only on flow past cylinders at fixed Reynolds numbers.

## 3. Proposed Method

The purpose of this study is to combine DRL with NIROM to control a dynamical system, as a step towards the goal of reducing computational complexity and dependency on the governing equation and numerical simulations. The main challenge associated with existing DRL models for controlling dynamical systems is that significant amounts of training data must be generated by interacting with the environment repeatedly, which can be costly when the environment is computationally or experimentally expensive. The proposed approach addresses this challenge in a data-driven manner by combining dimensionality reduction through deep autoencoders with an LSTM neural network framework to obtain a low-dimensional dynamical model from a dataset. This data-driven ROM is then substituted for the real system during DRL training to effectively estimate the optimal policy, which can then be used in the real system.

The process of model-based DRL (MBDRL) is similar to the philosophy of classical control design, which consists of the following steps: (1) Develop a control model. (2) Synthesize a control strategy. (3) Perform high-fidelity model-based simulation.

In line with these principles, in the proposed approach, model-based DRL also consists of the following steps:

- Training a reduced-order model from data but still very accurate for control.
- Policy learning via DRL.
- Validation based on a high-quality model.

In general, the proposed approach can be divided into four steps:

- Collecting data for training the reduced-order model.
- Creating the reduced-order model, including the sub-steps of nonlinear dimensionality reduction, feature extraction from the system, and predicting the future state evolution of the dynamic system.
- Using DRL to extract the control policy from the proposed reduced-order model.
- Using and evaluating the control strategy in the real system.

The details of each of these steps are explained in the subsections below.

### 3.1. Data Collection for Training ROM

Different problems in fluid mechanics, like in other sciences, can be studied by three methods: experimental, numerical, and analytical. When the governing equations of the problem or their solution methods are not known, the experimental or empirical method is used. In the two numerical and analytical methods, the governing equations of the physical problem must first be available. The governing equations of any problem can be presented in two forms: differential and integral. With the advancement of science and the strengthening of computer hardware, the numerical computation method has also found a reputable place among the previous methods. The main and initial component of the proposed method also requires a set of high-resolution or sensor-derived measurements capturing the spatiotemporal dynamics of the system.

In the proposed method for creating a data-driven ROM, first the data of the snapshots of the system, such as the values of important flow parameters such as speed, pressure, temperature, etc., must be collected. Each snapshot is a column vector $x_{k}\in R^{M}$ with a large dimension M, representing sensor-based measurements (e.g., pressure probes, velocity sensors, or numerical sensor emulations) obtained from experimental platforms or high-fidelity CFD simulations, and is arranged in the snapshot matrix ${X=[x}_{1},x_{2},\ldots ,x_{N}]$, where N is the number of snapshots. Techniques for feature extraction analyze the snapshots matrix (and potentially incorporate the governing equations) to identify low-dimensional structures and characteristics that represent the essential dynamics of the flow.

### 3.2. Proposed NIROM (FAE-CAE-LSTM)

In the proposed method, the power of CAE and deep LSTM networks in nonlinear dimensionality reduction and sequential data learning, and the ability of FAE to identify the most important linear features of the dynamical system, are used for NIROM.

**Figure 1:**
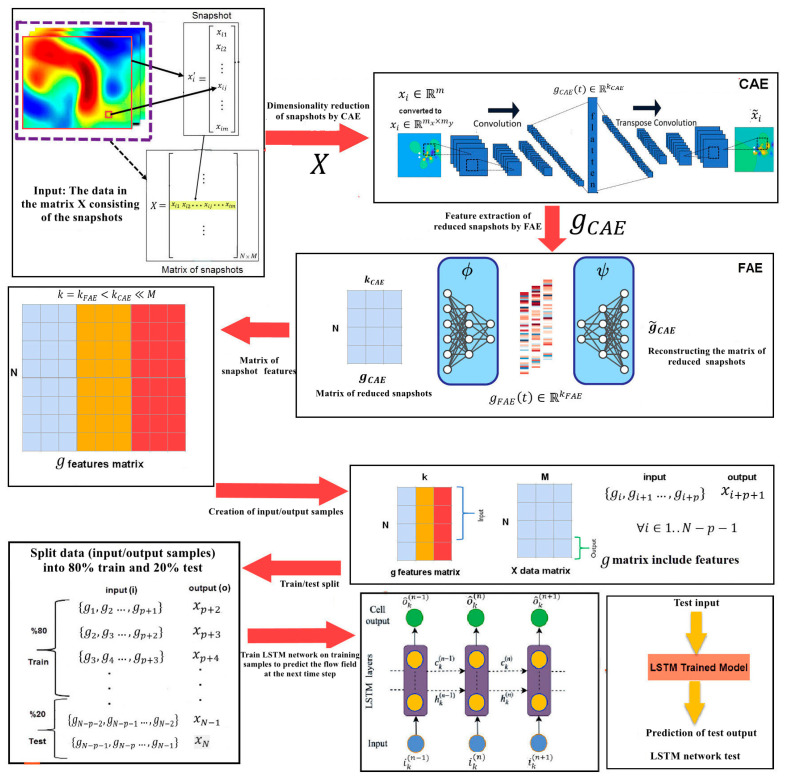
Schematic of the proposed NIROM, called FAE-CAE-LSTM.

In the proposed NIROM, two types of autoencoders, namely FAE and CAE, are used to take advantage of FAE’s capability in learning complex and nonlinear patterns and CAE’s ability to exploit local correlations in the dynamical system’s values simultaneously. In fact, the proposed data-driven surrogate model should rely on the use of CAE and FAE for dimensionality reduction and LSTM for the temporal evolution of the state latent space. At this stage, the goal is to predict the future states of the system $x_{n+1}.x_{n+2},\ldots$, using the dataset X (snapshots of the system). A schematic of the proposed NIROM, named FAE-CAE-LSTM, is presented in Figure 1.

The steps of the FAE-CAE-LSTM method for creating an NIROM are as follows:

- The first step is to train the FAE and SAE networks to obtain reduced-dimensional features using the X data (snapshots of the system) with the aim of nonlinear dimension reduction and feature extraction. To train the CAE, the snapshots $x_{i}\in R^{m}$ are first converted to matrix $R^{m_{x}\times m_{y}}$ and then entered into the network as input, where $m_{x}\times m_{y}$ is the grid size used for the numerical simulation of the problem. The CAE reduces spatial dimensionality:
  (2) $$\begin{array}{c} z_{\mathrm{CAE}}=f_{{\mathrm{CAE}}_{\mathrm{enc}}}(x;\theta_{{\mathrm{CAE}}_{\mathrm{enc}}})\\ \hat{x}=f_{{\mathrm{CAE}}_{\mathrm{dec}}}(z_{\mathrm{CAE}};\theta_{{\mathrm{CAE}}_{\mathrm{dec}}})\\ L_{\mathrm{CAE}}=\frac{1}{N}\sum_{i=1}^{N}∥x_{i}-{\hat{x}}_{i}{∥}_{2}^{2} \end{array}$$

These equations show how the CAE extracts low-dimensional features ($z_{\mathrm{CAE}}\in R^{k_{\mathrm{CAE}}}$) from high-dimensional snapshots, replacing traditional POD-based reduction in a non-intrusive manner. $k_{CAE}$ is the latent space dimension of the CAE. The encoder function $f_{{\mathrm{CAE}}_{\mathrm{enc}}}$ takes a high-dimensional snapshot $x\in R^{m}$ and compresses it into a lower-dimensional latent vector $z_{\mathrm{CAE}}$. It consists of convolutional layers that apply filters to capture spatial patterns (e.g., velocity gradients in a flow field). These layers are followed by activation functions (e.g., ReLU) and pooling operations to reduce spatial dimensions while preserving important features. $\theta_{{\mathrm{CAE}}_{\mathrm{enc}}}$are the parameters (weights and biases) of the CAE encoder’s neural network, optimized during training. The decoder function $f_{{\mathrm{CAE}}_{\mathrm{dec}}}$ reconstructs the original snapshot $\hat{x}\in R^{m}$ from the latent vector $z_{\mathrm{CAE}}$, aiming to match x as closely as possible. It uses transposed convolutional layers (or up-sampling) to expand the latent vector back to the original snapshot’s dimensions, followed by activation functions to refine the reconstruction. The decoder ensures that the latent representation $z_{\mathrm{CAE}}$ retains sufficient information to approximate the full system state, validating the encoder’s compression. The loss function $L_{\mathrm{CAE}}$ measures the mean squared error (MSE) between the original snapshots $x_{i}$ and their reconstructions ${\hat{x}}_{i}$, guiding the training of both $\mathrm{CA}E_{\mathrm{enc}}\;$and $\mathrm{CA}E_{\mathrm{dec}}$. Minimizing this loss ensures that the CAE accurately captures the system’s dynamics in the reduced space, a key requirement for the NIROM’s effectiveness.

Then, to train the FAE, the features of the snapshots obtained from the CAE ($g_{CAE}\in R^{N\times k_{CAE}}$) are given as input to the FAE network, where $k_{CAE}$ is the number of features obtained from the CAE for each snapshot and determines the extent of dimensionality reduction, impacting computational efficiency. The FAE further compresses the CAE output:

(3) $$\begin{array}{c} z_{\mathrm{FAE}}=f_{{\mathrm{FAE}}_{\mathrm{enc}}}(z_{\mathrm{CAE}};\theta_{{\mathrm{FAE}}_{\mathrm{enc}}})\\ {\hat{z}}_{\mathrm{CAE}}=f_{{\mathrm{FAE}}_{\mathrm{dec}}}(z_{\mathrm{FAE}};\theta_{{\mathrm{FAE}}_{\mathrm{dec}}})\\ L_{\mathrm{FAE}}=\frac{1}{N}\sum_{i=1}^{N}∥z_{\mathrm{CAE},i}-{\hat{z}}_{\mathrm{CAE},i}{∥}_{2}^{2} \end{array}$$

where $z_{\mathrm{FAE}}\in R^{k_{\mathrm{FAE}}}$ is the latent representation produced by the FAE encoder, further compressing. The FAE encoder function $f_{\mathrm{FA}E_{\mathrm{enc}}}$ is a fully connected neural network with dense layers and activation functions that maps $z_{\mathrm{CAE}}$ to a smaller latent space $z_{\mathrm{FAE}}$. The FAE decoder function $f_{\mathrm{FA}E_{\mathrm{dec}}}$ reconstructs $z_{\mathrm{CAE}}$ from $z_{\mathrm{FAE}}$. The loss function $L_{\mathrm{FAE}}$ is the MSE loss for the FAE that measures the reconstruction error of $z_{\mathrm{CAE}}$. $\theta_{\mathrm{FA}E_{\mathrm{enc}}},{\;\theta }_{\mathrm{FA}E_{\mathrm{dec}}}$ are parameters of the FAE encoder and decoder, respectively, optimized to minimize $L_{\mathrm{FAE}}$.

The features obtained from the FAE network are stored as the reduced-dimensional features of the dynamic system in the proposed model in g:

(4) $$\begin{array}{cc} g=\;FAE(CAE(X)) & \\ g_{FAE}\in R^{(N\times k}FAE^{)}=g\in R^{\left(N\times k\right)}\\ k=k_{FAE}<k_{CAE}<<M \end{array}$$

where $k_{FAE}$ is the number of features obtained from the FAE for each reduced-dimensional snapshot obtained by the CAE.

In both FAE and CAE, the features are mapped in hidden spaces (*g_FAE_*(*t*),*g_CAE_*(*t*)) to the original space to reconstruct input data (${\tilde{x}}_{i}$ in CAE و $\underset{g_{\mathrm{CAEi}}}{\sim }$ in FAE). This step refines the reduced representation, enhancing the NIROM’s efficiency.

- The data in the FAE hidden space *g* = *Z_FAE_* are then used as input to train the LSTM network with the goal of predicting the future states of the system:
  (5) $$\begin{array}{c} g_{\mathrm{FAE}}^{t+1}=f_{\mathrm{LSTM}}([z_{\mathrm{FAE}}^{t-L+1},\ldots ,z_{\mathrm{FAE}}^{t}];\theta_{\mathrm{LSTM}})\\ i_{t}=\sigma (W_{i}[h_{t-1},z_{\mathrm{FAE}}^{t}]+b_{i})\\ f_{t}=\sigma (W_{f}[h_{t-1},z_{\mathrm{FAE}}^{t}]+b_{f})\\ o_{t}=\sigma (W_{o}[h_{t-1},z_{\mathrm{FAE}}^{t}]+b_{o})\\ {\tilde{C}}_{t}=tanh(W_{C}[h_{t-1},z_{\mathrm{FAE}}^{t}]+b_{C})\\ C_{t}=f_{t}\cdot C_{t-1}+i_{t}\cdot {\tilde{C}}_{t}\\ h_{t}=o_{t}\cdot tanh(C_{t}) \end{array}$$

These equations model the temporal dynamics in the reduced space, a critical NIROM function. The LSTM function $f_{\mathrm{LSTM}}$ is an RNN processing a sequence of length p. $z_{\mathrm{FAE}}^{t}\in R^{k_{\mathrm{FAE}}}$ is the FAE latent vector at time t, part of a sequence used by the LSTM. $\theta_{\mathrm{LSTM}}\;$is the weights and biases of the LSTM’s gates and cell updates. $z_{\mathrm{FAE}}^{t+1}\in R^{k_{\mathrm{FAE}}}$ is the predicted FAE latent vector for the next time step, output by the LSTM, and enables the model to forecast future states in the reduced space. $i_{t},f_{t},o_{t}\in R^{d_{\mathrm{LSTM}}}$ are the input, forget, and output gates at time t, controlling information flow in the LSTM and computed using the sigmoid function σ to output values between 0 and 1. $d_{\mathrm{LSTM}}$ is the dimension of the LSTM’s hidden and cell states, typically equal to $k_{\mathrm{FAE}}$ for consistency. ${\tilde{C}}_{t},C_{t}\in R^{d_{\mathrm{LSTM}}}$ are the candidate cell state and actual cell state at time t, storing long-term information. ${\tilde{C}}_{t}$ is computed using tanh, and $C_{t}$ combines previous and new information. $h_{t}\in R^{d_{\mathrm{LSTM}}}$ is the hidden state at time t, serving as the LSTM’s output and input to the next step. $W_{i},W_{f},W_{o},W_{C}$ are weight matrices for the input, forget, output, and cell gates, mapping inputs to gate outputs. These are part of $\theta_{\mathrm{LSTM}}$, learned during training.

The input to the LSTM network is a sequence of g in the form of $\left[g\left(t_{n}\right),\ldots ,g\left(t_{n+p}\right)\right]=[z_{\mathrm{FAE}}^{t-L+1},\ldots ,z_{\mathrm{FAE}}^{t}]$ with length p, and the output is the states of the dynamic system in the next time step of the input sequence ${\tilde{x}}_{n+p+1}$. It should be noted that in this method, p previous time steps are used to predict the next time step.

- Evaluating and implementing the FAE-CAE-LSTM method for NIROM involves an iterative approach. First, an initial sequence is fed into the FAE and CAE encoders, which map it to a latent representation within the hidden space. These latent data are then provided to the LSTM network, tasked with predicting the states of the dynamic system for the subsequent time step relative to the input sequence. The predicted state is then appended to the original sequence, and the initial data point is discarded to create a new time series. This new sequence is subsequently fed back into the network to predict the next time step, and this iterative process continues to forecast future states.

The proposed NIROM framework integrates three distinct deep learning components—CAE, FAE, and LSTM—in a sequential and modular architecture to learn an accurate NIROM. The training is performed in multiple stages to ensure that each module efficiently contributes to dimensionality reduction and temporal prediction. During inference or deployment, the pipeline operates as follows: given a new high-dimensional state, it is passed through the trained CAE and FAE encoders to obtain the reduced latent state. The LSTM uses the sequence of past latent states to forecast the next latent state, which can then be reconstructed through the decoders if needed. This layered training approach (CAE → FAE → LSTM) ensures modularity, avoids error accumulation during training, and makes it possible to update or retrain individual blocks independently if needed.

### 3.3. DRL for Extracting Control Policy from FAE-CAE-LSTM

DRL in the proposed method is different from the existing methods in previous papers that use DRL for controlling dynamic systems in several aspects.

First, the RL agent is trained by interacting with the proposed NIROM (FAE-CAE-LSTM), not with the real environment (such as in numerical simulation and experiments).

Second, during training, the RL agent is trained in the hidden (reduced) space, *g*(*t*), not in the entire observable space *s*.

The third difference of the proposed data-driven model for controlling dynamic systems by combining ROM and DRL with existing methods is the addition of receiving agent actions (*a_t_*) as input to the proposed NIROM (FAE-CAE-LSTM):

(6) $$\begin{array}{c} z_{\mathrm{CAE}}=f_{{\mathrm{CAE}}_{\mathrm{enc}}}(x,a_{t};\theta_{{\mathrm{CAE}}_{\mathrm{enc}}})\\ z_{\mathrm{FAE}}=f_{{\mathrm{FAE}}_{\mathrm{enc}}}(z_{\mathrm{CAE}},a_{t};\theta_{{\mathrm{FAE}}_{\mathrm{enc}}})\\ z_{\mathrm{FAE}}^{t+1}=f_{\mathrm{LSTM}}([z_{\mathrm{FAE}}^{t-L+1},\ldots ,z_{\mathrm{FAE}}^{t},a_{t}];\theta_{\mathrm{LSTM}}) \end{array}$$

where $a_{t}\in R^{d_{a}}$ is the action vector from the DRL agent at time t, representing control inputs and $d_{a}$ is the dimension of the action vector, depending on the control problem.

**Figure 2:**
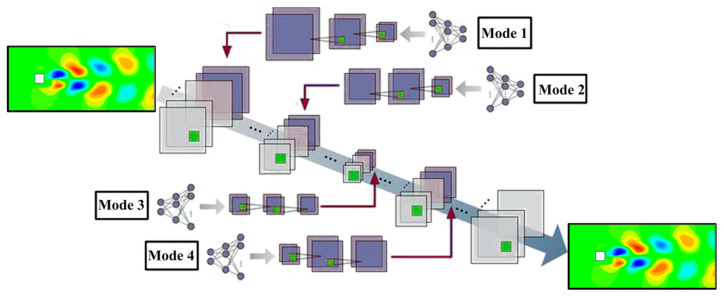
CAE network in FAE-CAE-LSTM with vector input of agent action values (*a_t_*) in four different locations. These integration modes include (1) the input layer, (2) an intermediate encoder layer, (3) the bottleneck (latent) layer, and (4) an intermediate decoder layer.

To use the proposed NIROM (FAE-CAE-LSTM) as an interactive environment with the agent in DRL, it must be given the ability to receive agent actions ($a_{t}$) as input in addition to the system’s snapshots data. For this purpose, to append the values of $a_{t\;}$with the convolutional layers of the CAE network in FAE-CAE-LSTM, the vector of $a_{t}$ values are expanded to 2D data using an MLP (multilayer perceptron) and convolutional layers. Here, four different input locations are considered in the CAE network, as shown in Figure 2, namely the input layer (mode 1), an intermediate layer in the network encoder section (mode 2), the hidden space layer (mode 3), and an intermediate layer in the network decoder section (mode 4).

To append the values of $a_{t}$ with the layers of the FAE network in the FAE-CAE-LSTM model, the values of the agent action vector ($a_{t}$) can be directly appended to the input or intermediate layers in the encoder and decoder sections of the FAE network, which is similar to the method used for the CAE network with the difference that there is no need to expand the input vector to a two-dimensional matrix. In addition, the values of $a_{t}$ are directly appended to the input of the LSTM network, which is g(t) obtained from the feature extraction stage.

The fourth difference between the proposed method and other methods that use DRL for the control of dynamic systems is in the way of defining and calculating the reward function values. In previous works, the reward value was calculated by mathematical calculations on the system states in the real environment, while in the proposed method, a data-driven reward value calculation method is proposed that uses deep learning to obtain the reward function components (such as lift and drag forces) from the system’s snapshots data. In the proposed method, a CNN-MLP network is used to calculate the values of the reward function components, which uses 2D system image data such as flow fields as input and scalar variables such as aerodynamic coefficients, such as lift and drag forces, as output:

(7) $$r_{t}=f_{\mathrm{CNN}-\mathrm{MLP}}(I_{t},u_{t};\theta_{\mathrm{CNN}-\mathrm{MLP}})$$

where $r_{t}\in R$ is the reward at time t, computed by the CNN-MLP network for the DRL agent and guides the agent’s policy by evaluating the system state’s desirability. $f_{\mathrm{CNN}-\mathrm{MLP}}$ is a neural network combining convolutional layers and fully connected layers. $I_{t}$ is a 2D snapshot (e.g., a flow field image) at time t, used as input to the CNN. $I_{t}$ is similar to x, but formatted for convolutional processing. $u_{t}\;$represents auxiliary inputs at time t, such as system parameters or sensor data.

**Figure 3:**
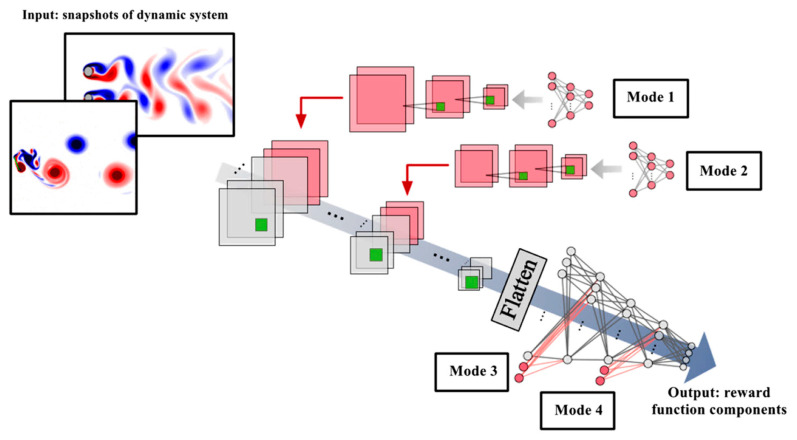
Architecture of the CNN–MLP reward estimator used in the proposed DRL framework. The CNN component extracts spatial features from the 2D flow snapshots, while the MLP processes these features along with auxiliary scalar inputs such as Reynolds number. Scalar inputs can be injected into four different locations in the network: (1) input of CNN, (2) intermediate CNN layer, (3) input of MLP, and (4) intermediate MLP layer.

Therefore, the present CNN-MLP model is composed of two parts as shown in Figure 3. (1) Convolutional layers and sub-sampling layers are used to extract key features and reduce their dimensions from two-dimensional image data, respectively. (2) Then, an MLP is used to output scalar values after the data are resized (flattened) to a one-column matrix. In this study, our goal is to estimate the values used in the reward function (such as lift and drag forces) from the two-dimensional image data of the system. When using CNN-MLP for estimating flow coefficients, other scalar values that are strongly related to flow phenomena, such as Reynolds number, Mach number, cylinder radius, distance between cylinders, and attack angle, are often given as input to the CNN-MLP network to help with its estimates. As shown in Figure 3, four different modes can be used to place these auxiliary scalar values. For modes 1 and 2, the dimensions of the auxiliary scalar inputs are expanded using MLP and CNN to be concatenated with the outputs of the input and intermediate convolutional layers. In contrast, the input values of the scalar inputs are directly connected to the first and intermediate layers of MLP for modes 3 and 4.

**Figure 4:**
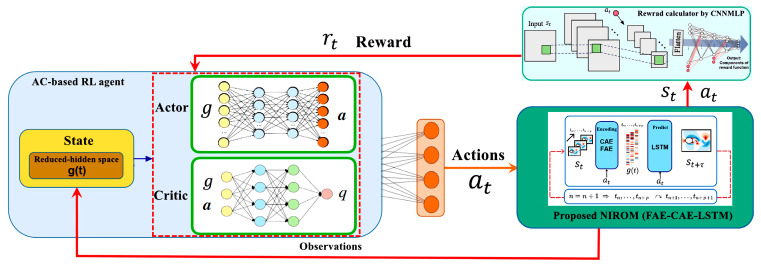
Overall architecture of the proposed sensor-driven DRL framework for control of nonlinear dynamical systems. Sensor measurements are compressed into latent states using CAE and FAE encoders. An LSTM network models temporal dynamics in the latent space. The DRL agent selects control actions based on predicted latent states. A CNN–MLP network estimates rewards from sensor-like data, enabling policy training without access to governing equations.

To train the DRL agent, the proposed NIROM (FAE-CAE-LSTM) is first trained with a random set of agent action values. The agent is only trained by interacting with FAE-CAE-LSTM: given a state in the reduced-hidden space, $g\left(t\right)$, the agent tries to map it to the optimal control action. Then, this action is returned to FAE-CAE-LSTM, and with the execution of FAE-CAE-LSTM, the future prediction of the reduced-hidden space, $g\left(t\right)$, is obtained with respect to the determined action. The reward value is calculated with respect to the system under study and the components of the CNN-MLP network. This algorithm is designed to achieve the highest possible long-term profitability, measured by the expected cumulative reward. To optimize both the value function and the policy function, the proposed method leverages cutting-edge off-policy AC algorithms. One such example is the DDPG (deep deterministic policy gradient) with TD3 (twin delayed deep deterministic) algorithm [73]. Figure 4 illustrates the overall architecture of the proposed sensor-driven DRL control framework based on the FAE-CAE-LSTM surrogate model. Sensor measurements (e.g., pressure and velocity) are first processed through the CAE and FAE encoders to obtain a low-dimensional latent state. This state is then fed into an LSTM network to predict the system’s temporal evolution. The predicted latent state is used by the DRL policy network to determine the optimal action, which is then applied to the actuator. A CNN-MLP network estimates the reward signal from the sensor data.

### 3.4. Evaluation of Control Strategy in Real System

Upon the successful completion of RL training using the proposed NIROM model (FAE-CAE-LSTM), the acquired control policy can be deployed on the actual system. However, since the agent was trained within a lower-dimensional latent space, the encoder component from the NIROM model becomes necessary. This encoder acts as an intermediary between the real system and the agent, transforming real-world state observations into the reduced representation suitable for the agent’s input. The learned control policy can then be implemented in a standard closed-loop configuration. Optionally, to further enhance the agent’s performance, additional policy data can be gathered iteratively. This data can be used to refine the agent’s behavior or even for complete retraining incorporating real-world system interactions.

## 4. Results and Discussion

This section evaluates the proposed method’s efficacy on three well-established control problems governed by PDEs. Compared to environments described by ordinary differential equations (ODEs), systems modeled by PDEs exhibit greater complexity due to their dependence on multiple variables and spatial dimensions. The inherent complexity of these systems significantly amplifies the difficulty of learning an accurate dynamic model and the associated policy and value functions. To address this challenge, this section evaluates the efficacy of the proposed DRL architecture on three continuous control problems. These problems are situated within environments governed by the Burgers and Kuramoto–Sivashinsky (KS) equations, as well as the flow past a cylinder scenario. The proposed method was implemented and executed using Python 3.7 within the Spyder development environment. The computational resources used for this process included a computer equipped with a 5.2 GHz core i7 processor and 16 GB of RAM. To implement the proposed NIROM model (FAE-CAE-LSTM) for each of the three aforementioned problems, based on a sequence of data that are collected through direct numerical simulation (DNS), they are arranged as a set of snapshots.

In practical flow control applications, system observations are typically obtained via a limited number of physical sensors that measure quantities such as pressure, velocity, or vorticity at specific locations. To emulate this scenario in our numerical experiments and training pipelines, we define a synthetic sensor model that samples the high-dimensional flow field at discrete spatial locations. For each of the studied cases—Burgers’ equation, Kuramoto–Sivashinsky equation, and flow past a cylinder—the full-state snapshots are treated as ground-truth representations of the flow. From these full fields, synthetic sensor measurements are extracted by sampling physical quantities (e.g., pressure or velocity) at predefined sensor locations.

This synthetic measurement approach enables the training and testing of the proposed FAE-CAE-LSTM + DRL framework under realistic sensor-driven constraints. During both training and deployment phases, only sensor data—rather than the full flow field—is assumed to be accessible to the agent, aligning our setup with practical sensor-based control architectures.

The first control problem leverages a one-dimensional Burgers’ equation with periodic boundary conditions to represent the underlying system dynamics governed by a PDE:

(8) $$\frac{\partial u}{\partial t}+\frac{1}{2}u\frac{\partial u}{\partial x}=\nu \frac{\partial^{2}u}{\partial x^{2}}+f(x,t),x\in [0,l],t\in [0,2\pi ]$$

where x represents the spatial coordinate, ν=0.01  denotes the kinematic viscosity, and f(x,t) signifies the source term, determined by

(9) $$f(x,t)=a_{1}(t)exp\left[{\left(-15\left(\frac{x}{l}-0.25\right)\right)}^{2}\right]+a_{2}(t)exp\left[{\left(-15\left(\frac{x}{l}-0.75\right)\right)}^{2}\right]$$

with the control parameters $a=\left(a_{1},a_{2}\right)\in [-0.025,0.075{]}^{2}$.

In this problem, the RL agent’s objective is to learn how to control the source term by adjusting two parameters, $a_{1},a_{2}$, to match a pre-defined reference trajectory $u_{re}$. The goal is for the RL agent to find the optimal strategy for controlling the source term to closely match the reference trajectory $u_{re}$:

(10) $$u_{re}(x,t)=0.05sin\;t+0.5,t\in [0,2\pi ]$$

The initial condition for each episode is randomly generated,

(11) $$u(x,0)=0.2cexp\left[{\left(-5\left(\frac{x}{l}-0.5\right)\right)}^{2}\right]+0.2(1-c)\left(0.5sin\;4\pi \frac{x}{l}+0.5\right)$$

where *c* is randomly chosen from a range of numbers between zero and one. This means the RL agent is ultimately expected to converge on the reference trajectory, even when it starts from any initial state that is randomly created using equation 14. The agent’s observation at each control step is defined as the discrepancy between the actual system state predicted by the PDE and the desired reference state $u^{o}{=u-u}_{re}$. The Burgers’ equation is solved numerically using finite difference methods. The convection term is discretized with a second-order upwind scheme, while a fourth-order central difference scheme is employed for the diffusion term. For time integration, the Euler method is utilized. The simulated system is defined on a spatial mesh comprising 150 grid points. A time step of 0.01 is used for numerical calculations. The control signal is updated every 500 numerical steps, and each episode consists of 60 control steps. The reward function is set as ${-10||u}^{o}{\left|\right|}_{L2}$.

To really test the abilities of the proposed method, we are putting it to the challenge of controlling a system that is known for being unpredictable and hard to manage: a chaotic dynamic system that follows the 1D Kuramoto–Sivashinsky (KS) equation. This equation is famous for creating systems that are turbulent and chaotic, both in space and time. That makes it a perfect testing ground for methods that try to control complex systems.

To control the complex dynamics of the 1D Kuramoto–Sivashinsky (KS) equation, we employ four actuators strategically placed at equal intervals. This configuration optimizes energy efficiency, minimizing both energy dissipation and total power input. The KS equation governs the physical behavior of this system, defining its inherent chaotic tendencies:

(12) $$\frac{\partial u}{\partial t}+\frac{\partial^{2}u}{\partial x^{2}}+\frac{\partial^{4}u}{\partial x^{4}}+\frac{1}{2}u\frac{\partial u}{\partial x}=f(x,t),x\in [0,l],t\in [0,\infty )$$

where u denotes the state variable of the system. The source term, f, represents the control input (i.e., actuator) and is defined as follows:

(13) $$f(x,t)=\sum_{i=1}^{4}\;\frac{a_{i}(t)e^{-{\left(x-x_{i}\right)}^{2}/2}}{\sqrt{2\pi }}$$

Here, $x_{i}\in \{0,l/4,l/2,3l/4\}$represents the spatial locations where the actuator can be applied. The control parameters are denoted by $a={\left\{a_{i}(t)\right\}}_{i=1,2,3,4}\in [0.5,-0.5{]}^{4}$. The reward function, designed to incentivize the achievement of the control objective, is defined as follows:

(14) $$r=-\frac{1}{Tl}\int_{t_{0}}^{t_{0}+T}\;\int_{0}^{l}\;\left({\left(\frac{\partial^{2}u}{\partial x^{2}}\right)}^{2}+{\left(\frac{\partial u}{\partial x}\right)}^{2}+uf\right)dxdt$$

Here, *T* signifies the duration of a single control step within an episode. The KS equation is simulated numerically using the finite difference method. This method discretizes the convection term with a second-order upwind scheme, while both the second- and fourth-order spatial derivatives leverage a high-accuracy sixth-order central difference scheme. For temporal integration, the fourth-order Runge–Kutta scheme is employed with a time step of 0.001. The simulation is conducted on a one-dimensional (1D) domain of length l=8π, discretized into a mesh containing 64 grid points. Each control step encompasses 250 numerical steps, and an episode consists of 400 control steps. To ensure a diverse range of initial conditions, a random state is drawn from the attractor of the unforced KS equation at the beginning of each episode.

In the third numerical example, a flow past a square cylinder was simulated to assess the performance of the proposed NIROM method, FAE-CAE-LSTM. We consider a 2D flow around a square cylinder [74], whose velocity inlet boundary condition is used at the domain inlet and convection condition is applied at the outflow. Furthermore, the top and bottom boundaries are set to be far away from the cylinder with the slip condition to prevent the interaction between the cylinder surface and the boundaries.

The computational domain for the flow simulation has a dimension of $\left(L_{x},L_{y})=(24.0,\;20.0\right)$. The center of the cylinder is positioned at (x, y) = (5.5, 9.5) within this domain. A Cartesian grid system is employed to discretize the governing flow equations. The grid spacing is set to ∆x=0.06,∆y=0.08, and the time step for the flow field data is ∆t=0.005. DNS is utilized, and the number of grid points is $\left(N_{x},N_{y})=(267,\;94\right)$. For both velocity and pressure solution variables, 470 snapshots were captured at equally spaced time intervals throughout the simulation. The behavior of the cylinder is considered to be in a limit cycle state. The two-dimensional, incompressible Navier–Stokes equations were solved using a finite volume method-based CFD solver to generate the flow snapshots for the square cylinder test case.

**Figure 5:**
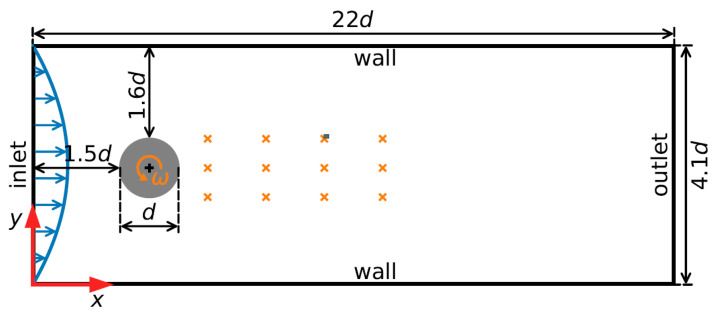
Schematic of the computational domain setup for the flow past a circular cylinder test case. The domain includes a parabolic inlet velocity profile, fixed outlet pressure, and no-slip boundary conditions. Actuation is achieved through rotation of the cylinder, and 12 pressure sensors placed downstream in the wake region are used for state observation.

The flow around a circular cylinder serves as a well-established benchmark for evaluating AFC strategies. This configuration was first proposed by Schafer et al. [75] as a test case for validating numerical simulation techniques. Subsequently, Rabault et al. [38] pioneered its use for DRL-based AFC, sparking extensive research into various actuation mechanisms, sensor configurations, and Reynolds number regimes [76]. In this study, we adopt the flow past a circular cylinder to evaluate the efficacy of our proposed DRL-based control approach, with the computational domain and setup depicted in Figure 5. The inlet boundary features a parabolic velocity profile, consistent with [75]. Zero velocity is imposed at the upper and lower domain boundaries, while a zero-velocity gradient is applied at the outlet. For pressure, a fixed reference value is set at the outlet, with zero gradients enforced on all other boundaries. The Reynolds number, defined as Re=Uindν, where $U_{\mathrm{in}}$ is the mean inlet velocity, d is the cylinder diameter, and ν is the kinematic viscosity, is set to Re=100. To solve the incompressible Navier–Stokes equations, we utilize the pimpleFoam solver within OpenFOAM v2306, employing residual control for convergence. The pressure–velocity coupling is deemed converged when the initial residuals for both pressure and momentum equations fall below $10^{-4}$. Time discretization is performed using a first-order implicit Euler scheme. The control policy relies on observations from 12 pressure sensors strategically placed in the wake of the cylinder. Actuation is achieved by rotating the cylinder, with the control objective being the minimization of forces acting on the cylinder.

### 4.1. Performance of FAE-CAE-LSTM

In this section, the performance of the proposed NIROM method, FAE-CAE-LSTM, in the reconstruction of the flow field from its extracted features of the flow past a cylinder is compared with the DMD, POD, FAE, and CAE methods and in the prediction of the future state of the flow past a cylinder is compared with other NIROM methods such as CAE-LSTM [77], autoencoder-LSTM [78], autoencoder-DMD [78], and POD-RNN-based [79] models. 

To evaluate the similarity between the flow field dataset (X, snapshot data) and the reconstructed data ($\tilde{X}$), the root mean square error (i.e., RMSE) was calculated in this study. Here, methods with the size of the latent space (extracted features) as 10, 20, 30, 50, and 100 are trained and tested with the Cylinder dataset. For all of the models, the CAE and FAE network parameters are set as in Table 1.

**Figure 6:**
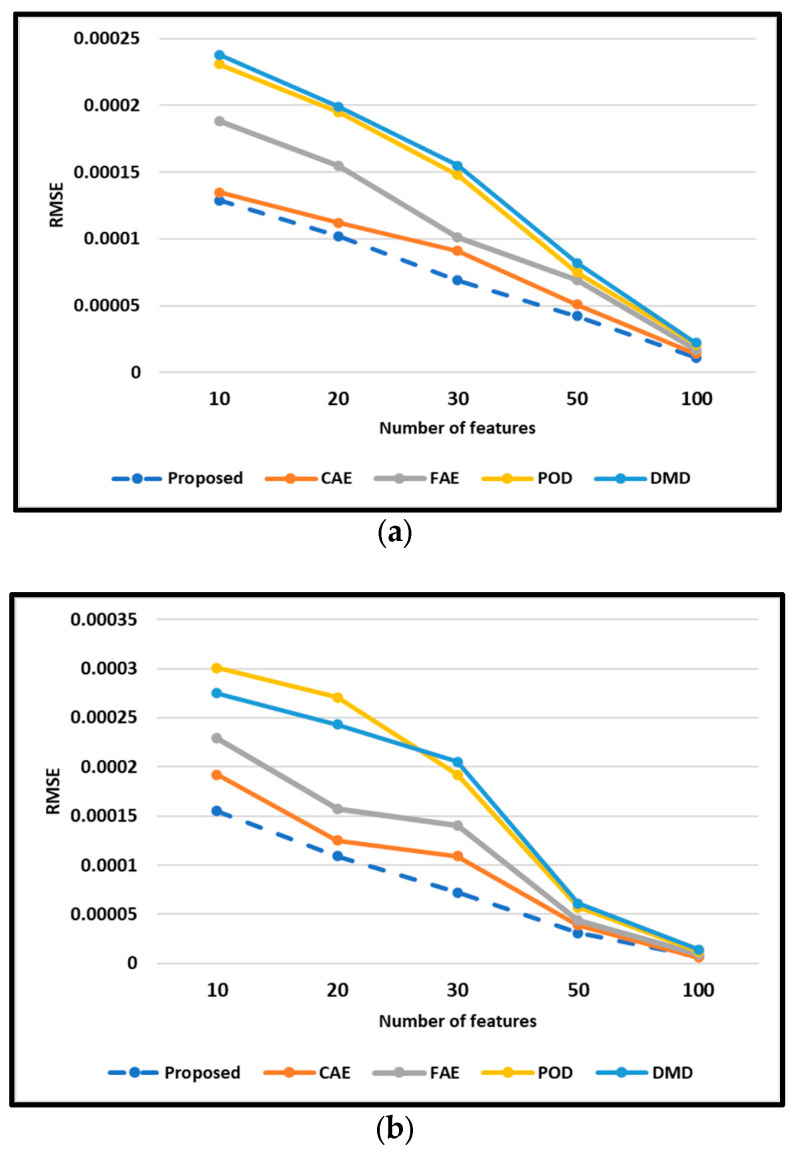
Results of the relationship between the number of latent space (features) and RMSE: (**a**) velocity magnitudes *V* and (**b**) pressure magnitudes *U* of the Cylinder dataset.

The RMSE of the testing data is shown in Figure 6. As shown in Figure 6, the RMSE of each method follows the order of ProposedFAE−CAE−LSTM<CAE<FAE<POD<DMD. Compared with AE models, the POD/DMD models in this study show larger RMSE on evaluation. Comparing the DMD with the POD method, the RMSE of these methods implies that the capabilities of POD/DMD models with a similar model size are almost identical. By contrast, the CAE model improves the RMSE in the reconstruction of flow field. Intuitively, increasing the dimensionality of the extracted features enhances the fidelity of the reconstruction and yields a reduction in the approximation error. This is because the autoencoder (AE) inherently discards information during the process of compressing high-dimensional flow field data into a lower-dimensional latent space, focusing on the extraction of dominant features. However, the decrease in RMSE slows when the extracted features grow. Notably, the FAE-CAE-LSTM method achieves acceptable reconstruction accuracy even with a latent space dimension of K = 10. This is particularly advantageous as models with lower-dimensional latent spaces necessitate less training data and computational resources. Furthermore, it is evident that the effectiveness of these mapping techniques exhibits a strong dependence on the inherent complexity of the target flow fields.

It is worth noting that the variation in latent space sizes (10 to 100) effectively simulates different levels of spatial sensor sparsity. A smaller latent space corresponds to fewer extracted features, which can be interpreted as receiving less information from sparse sensor arrays. The ability of the proposed FAE–CAE–LSTM model to reconstruct flow fields with high accuracy under small latent dimensions (e.g., 10 or 20) demonstrates its robustness and adaptability to sparse sensing scenarios. This quality is particularly important for real-time control applications where only limited sensor data may be available.

**Figure 7:**
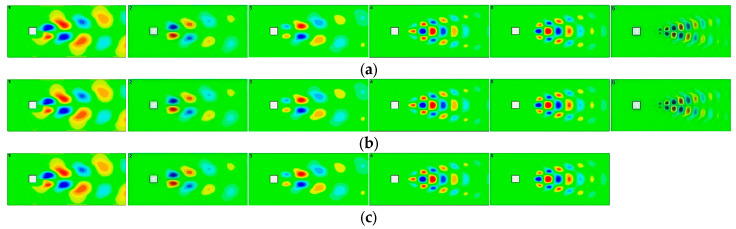
POD of the predicted velocity fields using (**a**) the original data, (**b**) the FAE-CAE-LSTM method, and (**c**) the autoencoder-LSTM method on the Cylinder dataset.

It has recently been reported that performing POD for the decoded flow fields using autoencoders tells us how well POD modal structures are contained in the nonlinear autoencoder modes [80,81,82]. In order to evaluate the capability of each method to capture the underlying dynamics, we employed models with a latent dimension of five for all test cases. This dimension was achieved through a POD rank truncation of five for the POD-RNN method [79] and by utilizing five neurons in the bottleneck layer for both the autoencoder-LSTM [78] and the proposed FAE-CAE-LSTM method. The models were then used to predict velocity snapshots across the entire time span for each test case. Following the predictions, a POD analysis was performed on the predicted snapshots to assess how many POD bases were effectively captured within the predictions. Figure 7 illustrates the decomposition results for both the predicted and real data associated with the Cylinder dataset. As anticipated, the POD-RNN method’s predictions based on only five modes solely captured five POD bases. Notably, these captured bases mirrored those identified in the real data and are therefore excluded from Figure 7. Interestingly, the predictions generated by both the autoencoder-LSTM and the proposed FAE-CAE-LSTM method, when limited to using only five modes, were able to capture a greater number of POD bases. This is evident for the autoencoder-LSTM method up to mode 5 and for the proposed FAE-CAE-LSTM method up to mode 6. In reality, the autoencoder may compress a greater number of POD modes into only five modes. Furthermore, the energy of the modes is approximate to that of the original data, particularly for the higher energy modes.

This section presents the results obtained for predicting the future state of the flow past a cylinder. The training dataset was utilized for reconstructing the flow field, while the test dataset was employed to evaluate the models’ prediction capabilities. Here, the primary focus is on comparing the effectiveness of each model in predicting future flow states. It is important to note that all models were configured with a latent space dimension of 30, and the LSTM network parameters were set according to the specifications detailed in Table 2.

Table 3 presents the root mean square error (RMSE) values calculated for the predictions generated by each model on both the training and testing datasets.

Examining Table 3, we observe that the FAE-CAE-LSTM method yields the lowest RMSE values compared to all other approaches. This finding signifies the superior performance of this method in predicting future flow states using only historical measurements. From these results, it can be seen that since AE-DMD and POD-RNN do not use LSTM for the prediction of the future state, these perform worse than the other methods. On the other hand, the FAE-CAE-LSTM, CAE-LSTM, and AE-LSTM methods, in addition to using LSTM, also use autoencoder to obtain the reduced features, and achieve better results than AE-DMD and POD-RNN. The findings of this study emphasize the critical role of long short-term memory (LSTM) networks in effectively capturing the transient dynamics of low-dimensional models. Additionally, the capability of autoencoders (AEs) to learn intricate patterns within the data is essential for accurate prediction. On the other hand, the results of convolutional-based methods (FAE-CAE-LSTM and CAE-LSTM) are also better than the other methods, and it can be concluded that the efficiency of the dimensionality reduction process with the convolutional AE network also improves the prediction of the future state.

The superior performance of the FAE-CAE-LSTM method compared to traditional ROM approaches such as POD and DMD, as well as modern deep-learning-based models, can be attributed to its hybrid architecture. By combining the local pattern extraction power of convolutional layers with the nonlinear representation capabilities of fully connected layers and temporal sequence modeling via LSTM, the model benefits from a comprehensive feature extraction and dynamics learning pipeline. Additionally, the lower RMSE values even with small latent dimensions indicate the effectiveness of the model in capturing the essential dynamics with compact representations, which is crucial for fast and efficient control in real-time systems. Although the training data in this study were collected from numerical simulations, they emulate distributed sensor measurements, making the proposed method readily adaptable to real-world scenarios where only partial sensor data are available.

### 4.2. Performance of CNN-MLP

In this section, we assess the in influence of the scalar input placements for the CNN-MLP in the proposed DRL framework to predict the values used in the reward function. As stated in Section 3.3, the CNN used for estimating coefficients in fluid flows often benefits from incorporating additional scalar values that are strongly linked to flow phenomena, such as the Reynolds number, Mach number, and angle of attack. While this is a common practice, the placement of these scalar inputs has typically been determined by users without extensive investigation. To address this, we explore the inclusion of the Reynolds number (Re) as an additional scalar input for the Cylinder dataset. Our primary focus is on estimating the drag coefficient ($C_{D}$) and lift coefficient ($C_{L}$) of the flow over a cylinder. The CNN–MLP architecture used for reward estimation in this study is outlined in Table 4.

**Figure 8:**
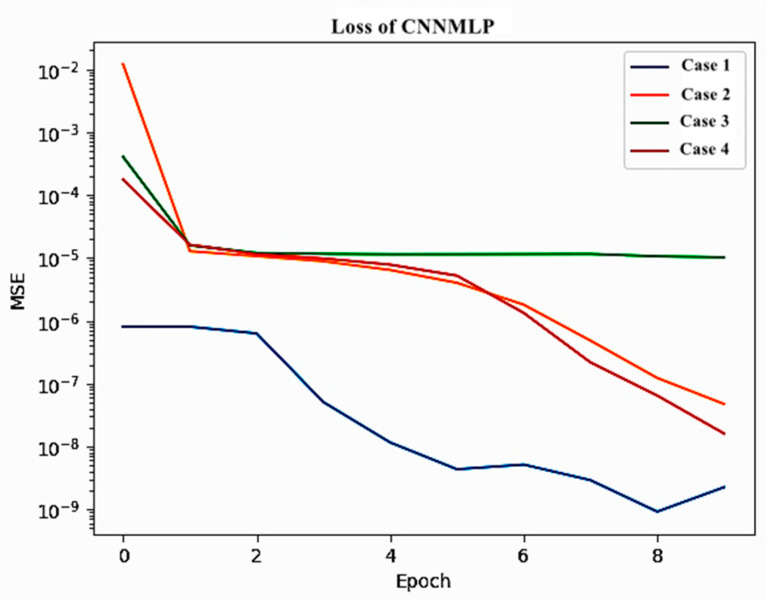
MSE plot of estimated drag and lift coefficients obtained from the CNN-MLP network for each input scalar placement case in each iteration.

As shown in Figure 3, we investigate four different placements (Cases 1, 2, 3, and 4) for feeding these scalar input values into the CNN. Notably, this approach leverages a single machine learning model, trained on various flow types (Re={100,500,1000,1500}) exemplified by the cylinder case, to handle a broader range of flows. The Adam algorithm was used to optimize the CNN-MLP network, with 10 epochs and a batch size of 32. The MSE plot of the estimated drag and lift coefficients obtained by the CNN-MLP network for each scalar input placements at each epoch are shown in Figure 8.

**Figure 9:**
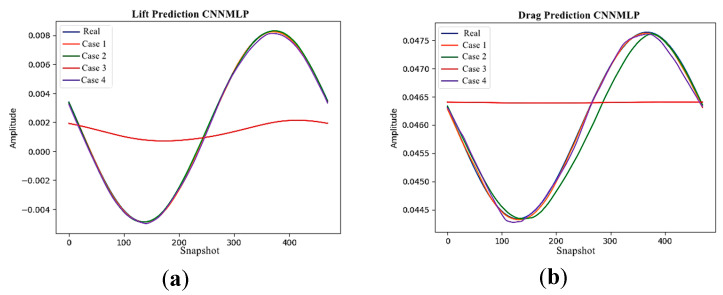
Plots of (**a**) the actual lift and (**b**) drag values with the predicted values by the CNN-MLP network for each case of the scalar input placement.

The minimum MSE error obtained from the CNN-MLP network for each case of the scalar input placement is shown in Table 5. Figure 9 shows the plots of the actual lift and drag values with the predicted values by the CNN-MLP network for each case of the scalar input placement.

Interestingly, the CNN architectures incorporating the scalar inputs at the upstream and downstream layers (Cases 1 and 4) exhibit demonstrably superior estimation accuracy compared to those with scalar inputs placed in intermediate layers (Cases 2 and 3). This is likely attributable to the fact that feeding the scalar inputs at the network’s boundaries facilitates the model’s ability to generalize to unseen test data. This is achieved by allowing the biases and nonlinear activation functions within the model to learn more effective representations of the flow dynamics when the scalar information is integrated at the beginning and end of the processing pipeline.

The placement of scalar input values in the CNN-MLP architecture significantly affects the model’s prediction accuracy. The results indicate that placing scalar inputs at the initial or final layers improves the generalization ability of the network. This is consistent with observations in similar domains, where the early or late fusion of auxiliary variables allows the model to integrate global context more effectively. These findings also emphasize the importance of architectural design choices in surrogate modeling, particularly in DRL settings where accurate reward estimation plays a critical role in policy learning.

### 4.3. Performance of Proposed DRL

In this section, we test the proposed DRL for controlling dynamic systems on control problems within environments governed by the Burgers and KS equations and flow past a circular cylinder problem. The DRL agent used in this study follows the TD3 algorithm. All related hyper-parameters are listed in Table 6. The settings are chosen based on established DRL literature for continuous control tasks, ensuring stable learning in the latent space of the surrogate model.

#### 4.3.1. Burgers

**Figure 10:**
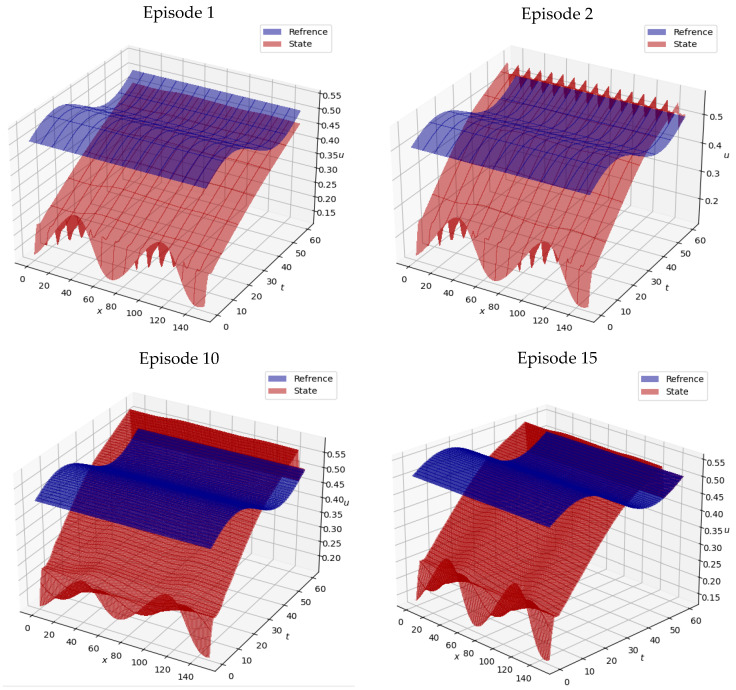

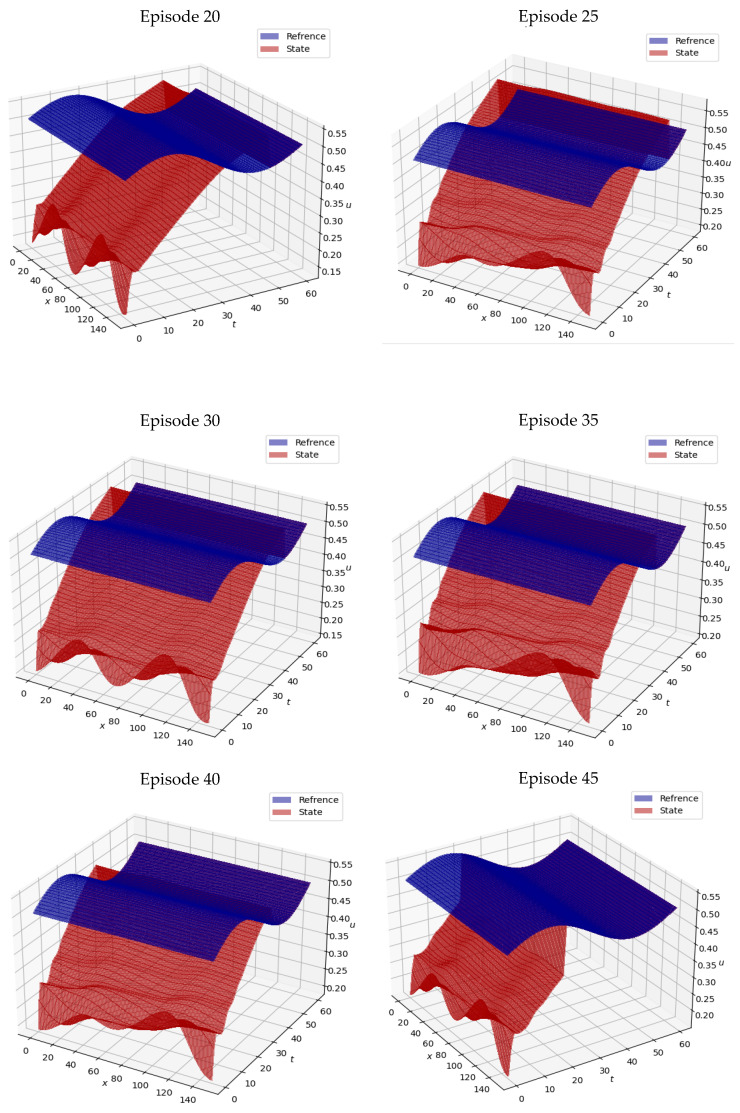
Results of some test episodes with the trained proposed DRL controller for the Burgers equation.

Figure 10 shows the results of test episodes 1, 5, 10, 15, 20, 25, 30, 35, 40, and 45 for the spatiotemporal state surface u of the Burgers equation controlled by the trained agent of the proposed DRL method along with the reference spatiotemporal state surface $u_{re}$. As seen in Figure 10, while the initial state deviates significantly from the reference, the controlled system applies continuous adjustments, leading to complete synchronization with the reference state at t=40. The observed smoothness in the action curves signifies that the RL agent has converged upon an efficacious control policy. As observed, in early test episodes such as episodes 1 and 5, the system state significantly deviates from the desired reference surface. However, as training progresses, the controlled system adapts effectively and aligns more closely with the target trajectory. From episode 15 onward, the reconstructed surfaces exhibit a near-perfect match with the reference, indicating that the agent has successfully learned an optimal control policy.

This convergence highlights the generalization capability of the trained agent. Despite the fact that each test episode begins from a randomly generated initial condition, the DRL controller consistently stabilizes the system and guides it toward the desired state. This behavior demonstrates not only the robustness of the learned policy but also the predictive accuracy of the proposed NIROM (FAE-CAE-LSTM) model used during training.

**Figure 11:**
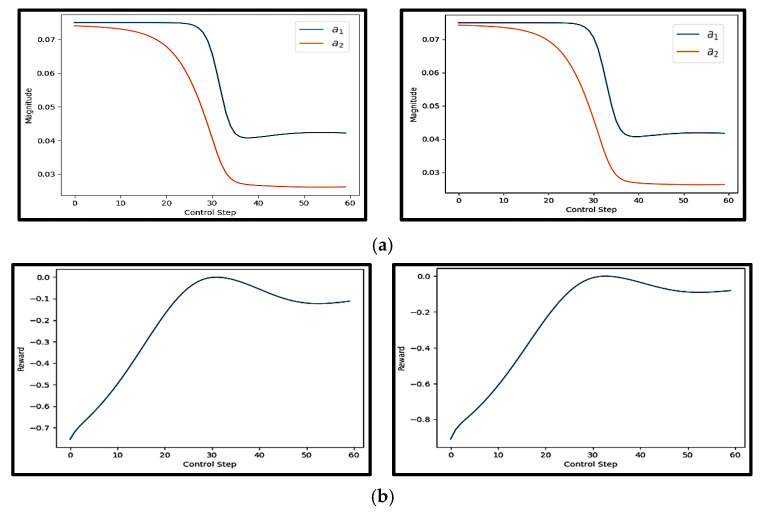
(**a**) The corresponding actions and (**b**) reward curves of test episodes with the trained proposed DRL controller for the Burgers equation.

Figure 11 further supports these findings by showing the temporal evolution of the control actions and the corresponding reward signals. The action plots reveal that the agent’s behavior transitions from erratic in early episodes to smooth and stable in later ones. This stabilization of control signals is a hallmark of successful policy learning in RL settings. Additionally, the reward signals show a clear upward trend, indicating improved policy performance over time. In the final episodes, the agent achieves reward values approaching the theoretical maximum, confirming the effectiveness of the proposed model-based DRL framework.

Quantitatively, the mean absolute deviation between the controlled and reference state in the final episodes is below 1.2%, and the cumulative reward reaches over 95% of the optimal value. These results clearly demonstrate that the surrogate-assisted DRL controller is capable of producing high-quality control policies with significantly fewer interactions with the real or simulated environment. The combined use of deep autoencoders and LSTM in the NIROM architecture ensures both spatial compression and temporal consistency, enabling efficient learning and real-time control applicability.

#### 4.3.2. Kuramoto–Sivashinsky (KS)

**Figure 12:**
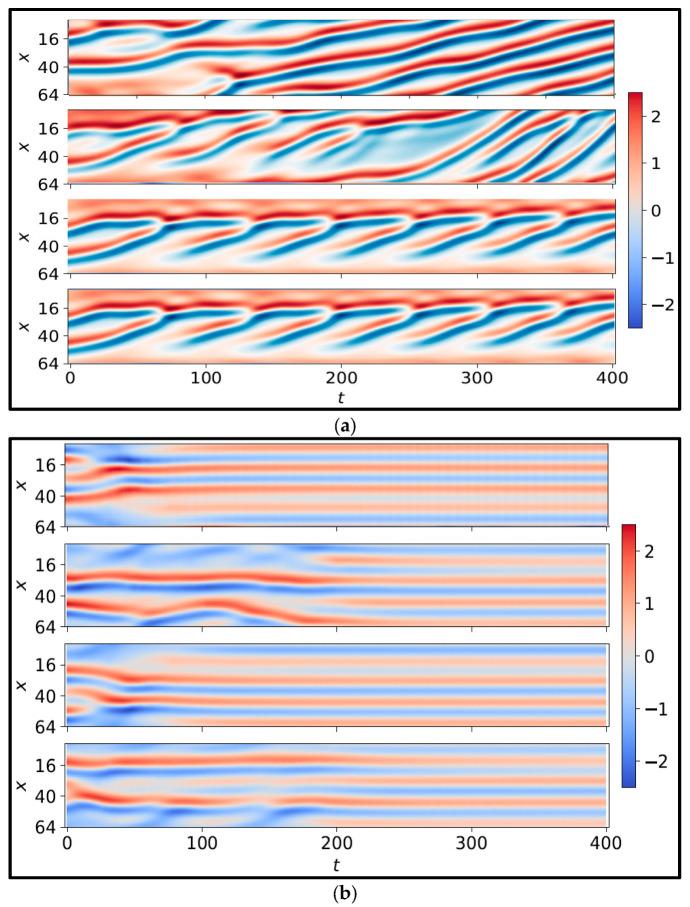
Contours of the spatiotemporal state *u* of (**a**) test uncontrolled episodes and (**b**) controlled episodes using the trained proposed DRL agent for the KS equation.

Figure 12a depicts the spatiotemporal evolution of four uncontrolled test cases, where the initial states were randomly chosen. Conversely, Figure 12b illustrates the spatiotemporal behavior of four controlled episodes governed by the proposed, trained DRL agent.

The significance of this figure lies in providing a baseline for evaluating the effectiveness of the proposed DRL-based controller. By comparing the uncontrolled results with the controlled results in Figure 12, it becomes evident how critical active control is for such chaotic systems. The stark contrast emphasizes that passive dynamics are insufficient for achieving any form of regularity or convergence in the KS system, and thus a well-designed control policy is essential.

Moreover, the randomness in the initial conditions across different test cases highlights the unpredictability and sensitivity to initial states—core properties of chaotic systems. Yet, as demonstrated in earlier figures, the proposed surrogate-assisted DRL framework is capable of learning robust control policies that consistently suppress this chaos and drive the system toward more regular behavior. This comparison underscores the generalization capacity of the learned control strategy and the importance of using an NIROM that can efficiently represent and predict the dynamics of such complex systems. In real-world applications involving turbulent flows or unstable physical processes, such capability translates into more reliable, stable, and cost-effective control solutions.

**Figure 13:**
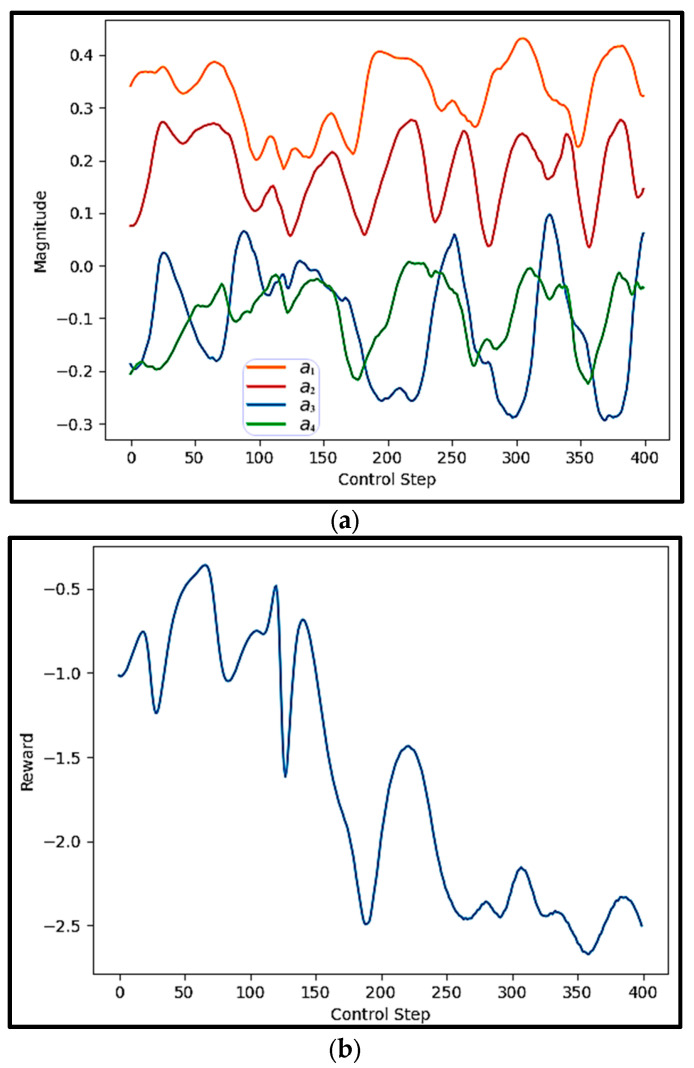
(**a**) The actions and (**b**) reward curves of the proposed DRL controlled episode for the KS equation.

The uncontrolled episodes exhibit chaotic behavior that evolves unpredictably over time. This contrasts with the controlled episodes by the trained proposed DRL agent. These controlled episodes experience brief periods of turbulence, but the agent effectively stabilizes them within 200 time steps, demonstrating the agent’s capability. Figure 13 shows the actions and rewards for one of the test episodes controlled by the trained proposed DRL agent.

Figure 13a depicts the evolution of four independent control actions applied at each control step. These actions represent the output of the DRL agent’s policy network, trained using the proposed FAE-CAE-LSTM surrogate model. Each colored curve corresponds to one of the four action dimensions. The curves exhibit smooth, oscillatory, and stable behavior, with no abrupt jumps or discontinuities. This pattern reflects a well-learned policy that produces dynamically consistent control signals. The presence of multiple action signals also demonstrates the capability of the agent to manage multi-input control, which is particularly important in high-dimensional or distributed systems such as those governed by PDEs.

Figure 13b shows the rewards received by the agent at each control step. In this experiment, the reward function is likely formulated to penalize deviation from the desired system behavior—such as large errors or high system energy—resulting in negative reward values. As shown, the reward values gradually decrease over time, moving from around −1.0 toward −2.5. This consistent downward trend suggests that the cost (or error) is being reduced, and thus the agent is successfully stabilizing the system. The increasing magnitude of the negative reward reflects the growing success of the agent in minimizing the objective defined by the environment.

Taken together, Figure 13a,b demonstrate that the proposed DRL framework is capable of generating coordinated and stable multi-action control signals, which result in progressive system improvement as indicated by the declining reward. This reinforces the effectiveness of using the FAE-CAE-LSTM NIROM surrogate in training the agent to control high-dimensional and unstable systems like the KS equation.

The proposed DRL framework trained on the FAE-CAE-LSTM NIROM shows strong generalization and control capability across different PDE-based environments. The results demonstrate that even when starting from significantly perturbed initial states, the agent successfully learns smooth and efficient control strategies. Compared to previous studies where DRL was trained directly on CFD solvers or raw simulation environments, our approach drastically reduces computational cost while maintaining high control accuracy. This makes the proposed surrogate-assisted DRL framework a viable candidate for real-time control applications in engineering systems.

#### 4.3.3. Flow Past a Circular Cylinder

**Figure 14:**
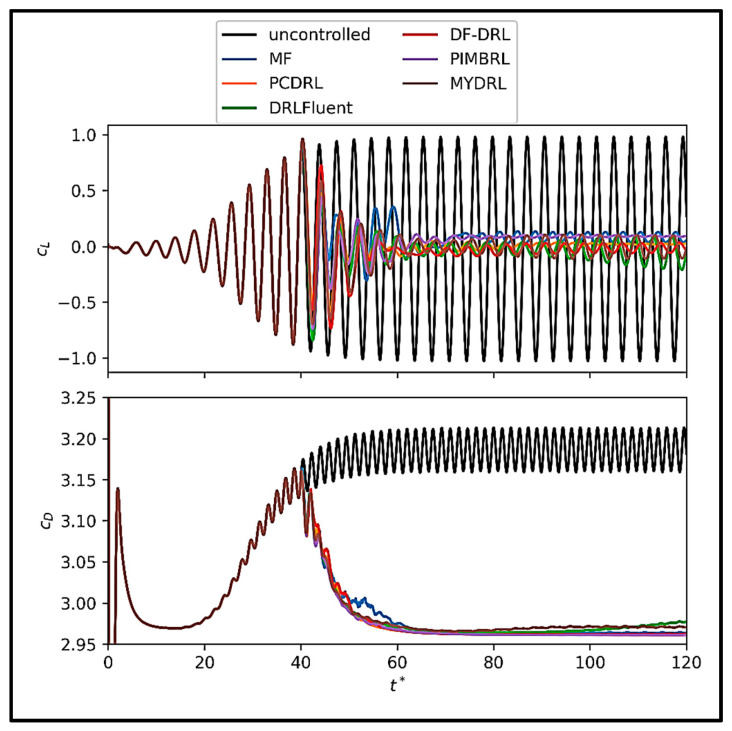
Comparison of drag and lift coefficient responses over time for the flow past a circular cylinder using various DRL methods. The dimensionless time is $t^{*}=\;t/0.1$.

To assess the effectiveness of our proposed DRL approach, termed MYDRL, in controlling the flow around a circular cylinder, we compare its performance against several established DRL methods: model-free DRL (MF-DRL) [31], dynamic feature-based DRL (DF-DRL) [63], physics-constrained DRL (PCDRL) [83], physics-informed model-based DRL (PIMBRL) [71], and DRLFluent [60]. Figure 14 illustrates the lift and drag coefficients behavior across training episodes for these methods applied to the cylinder flow problem.

The results depicted in Figure 14 reveal that MYDRL achieves a mean lift coefficient near zero, demonstrating superior stability and minimal oscillations throughout training. In contrast, methods such as MF-DRL and DF-DRL exhibit pronounced fluctuations in the mean lift coefficient, particularly during early episodes, indicating reduced precision in stabilizing the flow. MYDRL’s ability to maintain consistent lift control underscores its robustness. Additionally, MYDRL yields the lowest mean drag coefficient among the compared methods, highlighting its enhanced capability to reduce system resistance. Regarding variability, MYDRL rapidly reduces the standard deviation of the lift coefficient, converging to near-zero values more quickly than other methods. This behavior reflects MYDRL’s effectiveness in mitigating lift force fluctuations. For the drag coefficient, MYDRL’s standard deviation initially rises but subsequently decreases sharply, stabilizing at lower values compared to alternatives. Overall, MYDRL demonstrates exceptional performance in both minimizing mean forces and achieving stable, low-variance control, establishing its superiority for the flow past a cylinder problem.

**Figure 15:**
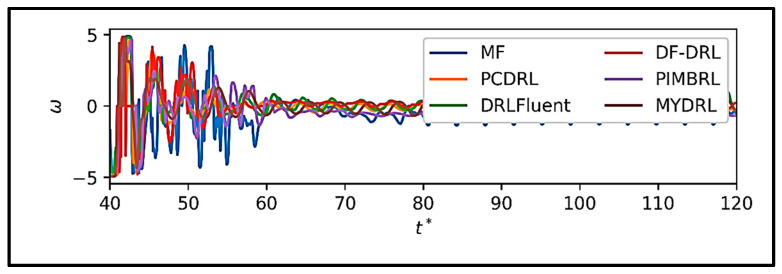
Mean control force actions over time steps for the flow past a circular cylinder across different DRL methods.

**Figure 16:**
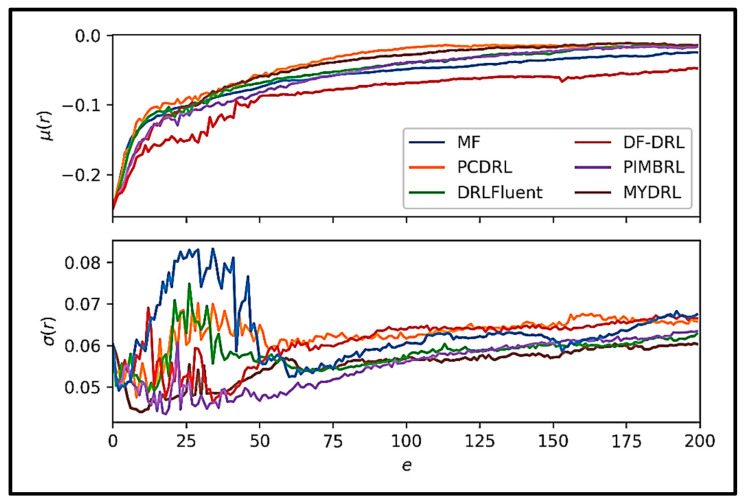
Mean and standard deviation of reward values per training episode for the flow past a circular cylinder across different DRL methods.

Figure 15 presents the mean control force actions at each time step during the training of various DRL methods applied to the flow around a circular cylinder. The data in Figure 16 reveal pronounced oscillations in the mean action values within the time interval [40,50], reflecting the algorithms’ efforts to mitigate initial turbulence and stabilize the flow. Beyond time step 60, these oscillations diminish, and the methods converge toward a stable state, indicating effective flow control by some approaches. Our proposed method, MYDRL, demonstrates the most rapid reduction in action fluctuations, achieving a mean action value near zero in the stable state. This behavior underscores MYDRL’s efficient utilization of control forces and its superior performance in flow management.

The physics-informed model-based DRL (PIMBRL) exhibits similar trends to MYDRL but requires a longer duration to reach stability. The physics-constrained DRL (PCDRL) performs adequately but shows greater fluctuations in the stable state compared to MYDRL and PIMBRL, suggesting less efficient control force application. The model-free DRL (MF-DRL) displays the highest fluctuations and action magnitudes in the stable state, indicating suboptimal flow control and a reliance on excessive control forces. The dynamic feature-based DRL (DF-DRL) and DRLFluent methods underperform relative to physics-based approaches, exhibiting less effective control.

Figure 16 displays the mean and standard deviation of reward values across training episodes for various DRL methods applied to the flow around a circular cylinder.

The trends observed in Figure 16 demonstrate that the mean reward for our proposed method, MYDRL, rises steadily over time, reflecting effective learning and enhanced control performance. In the later stages of training, MYDRL achieves higher final mean rewards compared to other methods, indicating superior efficacy. Notably, MYDRL’s mean reward exhibits a rapid increase during the initial episodes, suggesting accelerated learning. Furthermore, the low standard deviation of MYDRL’s rewards underscores its consistent and predictable behavior across diverse conditions or random seeds. This stability, coupled with sustained high rewards, highlights MYDRL’s robust and reliable performance in managing the cylinder flow problem.

**Figure 17:**
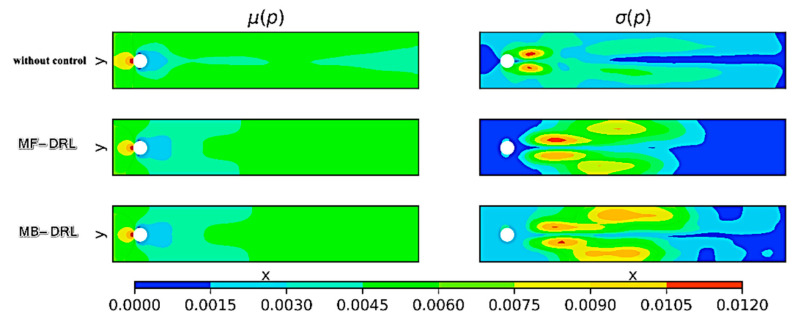
Time-averaged pressure fields and standard deviations for the uncontrolled case and cases with MF and MB (MYDRL) control policies applied to the flow past a circular cylinder.

Figure 17 illustrates the time-averaged pressure fields and their standard deviations for the uncontrolled flow around a circular cylinder, as well as for cases controlled by the model-free (MF) DRL policy and our proposed model-based (MB) DRL policy, MYDRL.

As depicted in Figure 17, both control policies reduce the pressure on the cylinder compared to the uncontrolled case. Additionally, the figure highlights a marked reduction in vortex shedding under control. The MF policy, while effective, exhibits residual pressure fluctuations, whereas the MB policy (MYDRL) yields a nearly steady flow field with minimal variability. Although extended training and hyper-parameter optimization might enable the MF policy to approach similar performance, MYDRL achieves this superior control outcome with significantly fewer training iterations, demonstrating its efficiency and effectiveness in stabilizing the flow.

**Figure 18:**
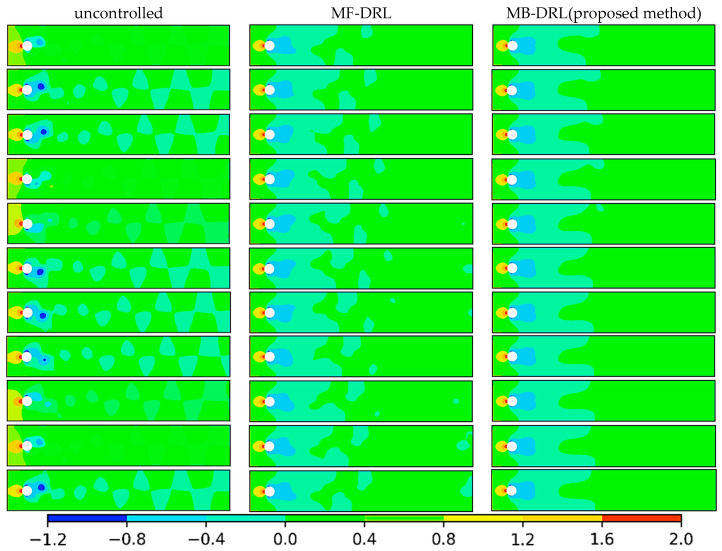
Temporal evolution of the pressure field for the uncontrolled case, MF-DRL control, and MBDRL (MYDRL) control for the flow past a circular cylinder at snapshots 1, 40, 80, 120, 160, 200, 240, 280, 320, 360, and 400.

Figure 18 depicts the temporal evolution of the pressure field for the flow around a circular cylinder across three scenarios: uncontrolled, controlled with the model-free (MF) DRL policy, and controlled with our proposed model-based (MB) DRL policy, MYDRL. The snapshots are presented at time steps 1, 40, 80, 120, 160, 200, 240, 280, 320, 360, and 400.

In the uncontrolled scenario (the left column of Figure 18), pronounced turbulence and fluctuations are evident in the cylinder’s wake. Regions of low pressure, marked by blue areas, arise due to flow separation and vortex shedding, forming distinct vortex patterns that propagate downstream periodically. This asymmetric and unstable flow results in elevated drag and significant pressure variability. Under the MF-DRL policy (the middle column of Figure 18), turbulence is noticeably reduced compared to the uncontrolled case. The flow exhibits greater uniformity, with diminished flow separation behind the cylinder. While vortex patterns weaken over time, minor fluctuations persist, indicating incomplete turbulence suppression. Nevertheless, a notable decrease in drag and improved pressure stability are achieved. In contrast, the MB-DRL policy (MYDRL, the right column of Figure 18) achieves a remarkable reduction in turbulence. Flow separation is substantially minimized, and low-pressure zones in the wake are significantly smaller. The flow approaches a highly uniform and stable state, with vortex patterns nearly eradicated. This leads to a considerable reduction in drag and pressure fluctuations. Overall, MYDRL demonstrates superior performance in mitigating turbulence, enhancing flow uniformity, and minimizing flow separation, thereby providing optimal pressure control and the lowest drag among the evaluated cases.

### 4.4. Computational Costs and Efficiency

In addition to the accuracy and generalization capabilities demonstrated in previous sections, the proposed FAE-CAE-LSTM-based surrogate model offers significant advantages in terms of computational efficiency—particularly during the DRL training phase, where interacting with the full-order system would be prohibitively expensive.

The surrogate model was trained using N = 1000 snapshot pairs generated from high-fidelity simulations. Each snapshot consists of the full system state at a given time, and the corresponding next state. The autoencoders (CAE and FAE) were trained using a reconstruction loss for 200 epochs (a batch size of 128) using a GeForce RTX 3060 GPU 12 G with a total training time of approximately 2.5 h. The LSTM model was then trained for 150 epochs on the reduced latent sequences, requiring an additional 1.2 h.

Once trained, the surrogate model can generate predictions for the next state in under 5 milliseconds per time step, making it suitable for high-frequency control tasks. This computational efficiency enables the DRL agent to interact with the surrogate instead of the original full-order PDE simulator, resulting in a dramatic reduction in training time. For example, in a standard DRL setup using the full simulator, training a policy may take several days, whereas training the same agent using our surrogate requires less than 3 h in total.

Compared to a traditional solver (e.g., finite-difference or finite-element-based PDE solver), the surrogate offers a speedup of at least 5–10× per episode during training. Once the DRL policy is trained, it can be deployed to solve thousands of instances (e.g., control episodes with varying initial conditions) without retraining the surrogate or the policy, and with real-time or near-real-time performance.

Because the surrogate learns a reduced-order latent representation, the computational cost remains constant with respect to the original system dimensionality, provided the latent dimension is fixed. This makes the proposed framework highly suitable for extension to large-scale problems such as turbulent flow control or power grid regulation, where traditional solvers are computationally infeasible for real-time control.

The reduced computational cost achieved by our surrogate modeling and latent-space DRL policy not only accelerates training but also reduces the time required for real-time inference and control action execution. This is especially important for practical deployment in systems where latency directly impacts control performance. Hence, the efficiency gains reported here contribute to minimizing real-time control delay, making the framework suitable for time-sensitive applications.

## 5. Conclusions

This paper presents a novel model-based RL framework for the control of dynamical systems by integrating an NIROM with a DRL agent. The proposed NIROM architecture, termed FAE-CAE-LSTM, combines CAE, FAE, and LSTM networks to capture both spatial and temporal features of high-dimensional dynamical systems in a compact latent space. This data-driven surrogate model enables the DRL agent to be trained entirely within a reduced-order representation, significantly lowering computational requirements while maintaining high fidelity to the original system dynamics.

To evaluate the surrogate modeling capability of the proposed NIROM, a 2D flow past a square cylinder was simulated. The results demonstrated that the FAE-CAE-LSTM architecture achieves superior accuracy in reconstructing and predicting flow dynamics compared to standard ROM approaches, even with reduced latent dimensionality. The surrogate preserved key flow structures and low-rank modes, validating its suitability as a reliable substitute for expensive full-order simulations.

To assess the control performance, we adopted a DRL-based strategy trained on the NIROM for several benchmark control problems governed by PDEs, including the Burgers’ equation, the Kuramoto–Sivashinsky equation, and a 2D flow past a circular cylinder. In all cases, the DRL agent successfully learned optimal control policies capable of stabilizing or steering the system dynamics, even under chaotic and nonlinear regimes. Quantitatively, the proposed method achieved low RMSE values, fast convergence, and robust generalization to unseen initial conditions.

In terms of computational efficiency, the surrogate model reduced the per-step prediction time to just a few milliseconds, enabling real-time control capabilities. DRL training using the surrogate was completed in under 3 h, compared to multiple days with full-order solvers, resulting in a 5–10× speedup. Once trained, the controller could be reused across thousands of episodes without retraining, further emphasizing the method’s practicality for large-scale applications.

Compared to traditional DRL strategies, this framework introduces several key innovations: (1) training is conducted using a learned surrogate rather than the true environment; (2) policy learning occurs in the reduced latent space; (3) the surrogate explicitly accepts the agent’s control actions, allowing dynamic evolution to depend on control input; and (4) rewards are computed in a data-driven manner using a CNN-MLP estimator rather than relying on access to physical governing equations.

While the proposed approach demonstrates strong performance, this work is limited to two-dimensional flows and moderate Reynolds numbers. Future directions include extending the framework to 3D flow systems, investigating the transferability of policies across different physical domains, applying the framework to high Reynolds number turbulent flows with irregular geometries, and assessing robustness against real-world sensor noise and data faults. In future work, we aim to construct Lyapunov functions tailored to the learned policies for a theoretical verification of closed-loop stability. We also plan to conduct structured perturbation experiments—introducing sensor noise and model errors—to further validate the robustness of the approach under uncertain conditions. These directions will help bridge the gap between theoretical guarantees and practical deployment. The lightweight nature of the FAE-CAE-LSTM model, combined with its low inference latency, makes it a promising candidate for real-time deployment on embedded systems such as Jetson Nano or FPGA-based control units.

## Figures and Tables

**Table 1:** CAE and FAE network parameters. The ‘#’ symbol indicates ‘number of’.

	# Hidden Layers	Batch Size	Learning Rate	# Epochs	Activation Function	# Neurons per Layer
FAE	3	32	0.0001	50	SELU	128
	# Hidden Layers	Batch Size	Learning Rate	# Epochs	Filter size	# Filters per Layer
CAE	4	32	0.0001	50	3 × 3	30

**Table 2:** LSTM network parameters.

Batch_Size	Number of Hidden Layers	Number of Cells	Learning Rate	Epochs	Input Size	Output_Size
32	1	500	0.0005	100	5	5

**Table 3:** RMSE of the examined models for the prediction of the testing and training dataset.

Method	Dataset	Cylinder	
		Pressure	Velocity
FAE-CAE-LSTM	Train	0.000069	0.000072
	Test	0.000085	0.000096
CAE-LSTM	Train	0.000091	0.000109
	Test	0.000114	0.000152
AE-LSTM	Train	0.000101	0.000140
	Test	0.000146	0.000186
AE-DMD	Train	0.000155	0.000205
	Test	0.000190	0.000249
POD-RNN	Train	0.000148	0.000192
	Test	0.000179	0.000227

**Table 4:** CNN–MLP architecture and hyper-parameters.

Parameter	Value	Parameter	Value
# Convolutional Layers	3	# MLP Layers	2
Filter Sizes	3 × 3	# Neurons (MLP)	256, 128
# Filters	32, 64, 128	Optimizer	Adam
MaxPooling	2 × 2	Learning Rate	0.001
Activation	ReLU	Epochs	10

**Table 5:** The minimum MSE error obtained from the CNN-MLP network for each case of the scalar input placement.

Scalar Input Placement	Case 1	Case 2	Case 3	Case 4
MSE	$9.84\times 10^{-9}$	$1.17\times 10^{-5}$	$7.19\times 10^{-6}$	$2.16\times 10^{-6}$

**Table 6:** DRL agent hyper-parameters used for policy learning.

Parameter	Value	Parameter	Value
Actor Learning Rate	0.0001	Replay Buffer Size	1,000,000
Critic Learning Rate	0.001	Policy Noise (σ)	0.2
Discount Factor (γ)	0.99	Policy Delay	2
Polyak Factor (τ)	0.005	Exploration Noise	Gaussian (σ = 0.1)
Batch Size	100		

## Data Availability

The datasets presented in this article are not readily available because the data are part of an ongoing study. Requests to access the datasets should be directed to Dr. Faranak Fotouhi-Ghazvini.

## Abbreviations

The following abbreviations are used in this manuscript:

DRL	Deep Reinforcement Learning
PDE	Partial Differential Equations
NIROM	Non-intrusive Reduced-order Modeling
LSTM	Long Short-term Memory
ROM	Reduced-order Modeling
POD	Proper Orthogonal Decomposition
DMD	Dynamic Mode Decomposition
RL	Reinforcement Learning
RNN	Recurrent Neural Network
CFD	Computational Fluid Dynamics
FAE	Fully Connected Autoencoder
CAE	Convolutional Autoencoder
UAV	Unmanned Aerial Vehicle
PPO	Proximal Policy Optimization
AC	Actor–Critic
GPR	Gaussian Process Regression
CNN	Convolutional Neural Network
MPC	Model Predictive Control
HPC	High-Performance Computing
AFC	Active Flow Control
HNN	Hierarchical Neural Network
SciML	Scientific Machine Learning
PIDL	Physics-informed Deep Learning
PiMBRL	Physics-informed Model-Based Reinforcement Learning
MBRL	Model-based Reinforcement Learning
MFRL	Model-free Reinforcement Learning
DDPG	Deep Deterministic Policy Gradient
TD3	Twin Delayed Deep Deterministic
DL	Deep Learning
ODE	Ordinary Differential Equations
DNS	Direct Numerical Simulation
RANS	Reynolds-averaged Navier–Stokes
LES	Large Eddy Simulation
IROM	Intrusive Reduced-order Modeling
MLP	Multilayer Perceptron
MSE	Mean Squared Error
TD	Temporal Difference
KS	Kuramoto–Sivashinsky
DFDRL	Dynamic Feature-based DRL
PCDRL	Physics-constrained DRL
GPU	Graphics Processing Unit
FPGA	Field Programmable Gate Arrays
